# Assessing the physical activity of parents of children suffering from cancer: a cross-sectional study

**DOI:** 10.1186/s12889-025-25455-5

**Published:** 2025-11-17

**Authors:** Carolin Ohnmacht, Charlotte M. Niemeyer, Antonia Pahl, Albert Gollhofer, Alexander Puzik

**Affiliations:** 1 Department of Pediatric Hematology, Oncology and Stem Cell Transplantation, Faculty of Medicine, Children’s Hospital, Medical Center – University of Freiburg, Freiburg, Germany; 2 Department of Sport and Sport Science, University of Freiburg, Schwarzwaldstraße 175, Freiburg, 79117 Germany; 3 Department of Medicine I (Specialties: Hematology, Oncology, and Stem-Cell Transplantation), Faculty of Medicine, Medical Center – University of Freiburg, Hugstetterstr. 55, Freiburg, 79106 Germany

**Keywords:** Physical activity, Childhood cancer, Parents, Sitting time, Long-term side effects, Physical complaints, Exercise program

## Abstract

**Background:**

Regular physical activity (PA) is essential for biopsychosocial health, but reduced PA during therapy for childhood cancer increases the burden of long-term side effects. Cancer and its therapy determine the everyday life of affected families, and parents spend plenty of time with their child in the hospital. Thus, we assumed that the restriction of the movement environment affects the parents’ PA-behavior. Meanwhile, parents have a pronounced influence on their child’s PA-behavior. Therefore, we investigated self-reported PA and sitting time (ST) of parents before and during their child’s cancer therapy.

**Methods:**

Forty parents provided their consent and participated in the study between September 2021 and February 2022. Parental PA and ST were reported before and during their child’s cancer therapy in a cross-sectional design using the International Physical Activity Questionnaire-Short Form (IPAQ-SF). The questionnaire was supplemented with questions dealing with attitude towards exercise, physical status of the parents and prerequisites for movement therapy. Analysis of variance was applied using IBM SPSS Statistics. The Ethics Committee of the University of Freiburg approved the study.

**Results:**

The self-reported parents’ PA-levels before their child’s diagnosis corresponded to reference values for healthy adults. During their child’s therapy, all reported dimensions of parental daily PA and the number of Metabolic Equivalent of Task (MET)-minutes of PA per week decreased significantly. Especially during inpatient stays, PA declined (mean ± SD: from 3004.5 ± 2301.4 to 889.1 ± 1585.1; *p* < 0.001), with a significant increase in ST in minutes per workday (mean ± SD: from 329.3 ± 196.6 to 687.8 ± 268.8; *p* < 0.001). In addition, 45% of the parents reported about new physical complaints since their child’s cancer diagnosis and 92.5% could imagine taking part in an exercise program.

**Conclusions:**

The findings of this retrospective, cross-sectional questionnaire study suggest that parental PA declines during a child’s cancer therapy. Nearly half of the parents reported new physical complaints since their child’s cancer diagnosis. To counteract these health hazards, future exercise programs in pediatric oncology should include parents to promote their own health and to enable them to act as role models for their children.

**Trial registration:**

German Register of Clinical Trials No.: DRKS00026248, prospectively registered on 17/09/2021.

**Supplementary Information:**

The online version contains supplementary material available at 10.1186/s12889-025-25455-5.

## Background

 Regular physical activity (PA) has numerous positive effects on human health [1, 2]. PA and an active lifestyle reduce the risk of cardiovascular disease [3], obesity and musculoskeletal disorders [2]. Regular exercise supports stress management and promotes mental health by improving personal and social resources, body image, mood and sleep quality [4, 5]. Additionally, PA alleviates depression and anxiety [6] and helps to maintain or improve overall fitness [2].

Despite the general awareness of the positive effects of PA, only few people are sufficiently active. For adults, the World Health Organization (WHO) recommends regular PA of at least 150–75 min per week (min/W) with moderate or vigorous intensity or an equivalent combination. To achieve additional health benefits, the WHO proposes strength training for the main muscle groups twice a week [2]. On the other side of the coin, the WHO states that physical inactivity is the fourth leading risk factor for all-cause mortality and is associated with the occurrence of non-communicable diseases (including cardiovascular disease, cancer and type 2 diabetes) [7]. A total of 55% of the German population do not reach the recommended levels of PA to improve and protect their health [8].

As PA decreases, sitting time (ST) (e.g. during work and leisure time) increases, resulting in the development of various chronic diseases and motor deficits [9–11]. Although physical inactivity is a known health risk, there are still uncertainties about the amount of sedentary time needed to increase disease risk and all-cause mortality.

When a family is confronted with a child’s cancer diagnosis, intensive and vital therapies, which are associated with several inpatient stays, suddenly determine everyday life [12]. During cancer therapy in childhood and adolescence, there is often a disease- and therapy-related restriction of the movement environment and thus a significant reduction in the level of activity. An overprotective attitude of the social environment increases inactivity [13]. In particular, during inpatient stays, affected children and adolescents experience a 91% reduction in their PA-level, whereby 50% leave their bed for less than one hour per day [14]. This can lead to amplification of therapy- and disease-related long-term side effects as cardiovascular diseases, obesity, chronic pain, orthopedic problems, fatigue, fear of recurrence or future and depression [15–17]. At the same time, parents have a lasting influence on their children’s PA-behavior, for example by acting as role models, being active themselves or together with their children [18–20].

Although there has been an increased expansion of sports therapy care structures for children and adolescents in German pediatric oncology centers in recent years, parents are not yet the focus. Because parents usually stay constantly at their child’s side during therapy, it can be assumed that not only the patients themselves, but also their parents are affected by therapy- and daily structure-related movement restrictions [21].

Physical and mental stress is common among family members of young cancer patients [12] and is associated with less PA and more sedentary behavior [4]. Stults-Kolehmainen and Sinha [4] show that stress not only reduces motivation to exercise, but also creates psychological barriers that make PA even more difficult. First studies show that parents of children with disabilities are significantly less active than parents of healthy children, due to increased caregiving demands [22].

Parental attitudes and stressors also play a crucial role in this context. During their child’s cancer therapy, many parents experience considerable emotional overload due to multitude of medical obligations and concerns about their child’s well-being [17, 23]. In addition, PA is often perceived as a secondary priority in everyday family life, or parents are unsure about the extent to which exercise plays a positive role during the treatment and rehabilitation process [24, 25]. PA protects from chronic diseases and psychological stress such as depression and anxiety disorders [4].

Despite the proven health-promoting effects of exercise, the physical inactivity of parents of chronically ill children has so far been insufficiently systematically investigated. Based on this background, this study aimed to assess the PA and ST of parents before and during their child’s cancer therapy using a questionnaire.

## Methods

This cross-sectional questionnaire study took place at the Department of Pediatric Hematology and Oncology at the Children’s Hospital of the Medical Center of the University of Freiburg in Germany. This study was approved by the Ethics Committee of the University of Freiburg and conducted according to the Declaration of Helsinki (German Register of Clinical Trials No.: DRKS00026248).

### Participants and procedures

Within a 6-month period (September 2021-February 2022), eligible parents were recruited at the pediatric oncology ward. Parents (18–65 years of age) with a child (< 18 years of age) who had been treated for cancer for at least four weeks or had been hospitalized for a second time of cancer treatment were included in the study. In addition, regular accompaniment of their child during inpatient stays and care at home by the corresponding parent were required. Exclusion criteria included insufficient understanding of the questionnaire and lack of consent form to the survey. The paper-based questionnaire, including all periods relevant to this study, was handed out once after informed consent was obtained on average 8.2 months since diagnosis (additional file 1: Table S1).

#### Physical activity (PA)

Intraindividual comparisons of parental PA before and during their child’s cancer therapy were assessed by the International Physical Activity Questionnaire-Short Form (IPAQ-SF) [26, 27]. The IPAQ-SF comprises seven items that measure the frequency (days per week), duration (minutes), and intensity (light, moderate and vigorous) of PA during an average week. For retrospective assessment, the parents surveyed were instructed to report PA during three different time periods: (1) before diagnosis, (2) during inpatient stays (hospital), and (3) during outpatient care (at home). The first section refers to a typical week before the onset of symptoms or the child’s diagnosis. The second section, records the PA during the child’s cancer treatment (each for inpatient and outpatient phases). It records self-reported activity e.g. at work or in hospital, in transportation (e.g. walking or cycling), in the household (including gardening) and during leisure time. For details please see original Activity-Questionnaire in appendix. For this primary endpoint, the metric outcome variable is the total activity in Metabolic Equivalent of Task in minutes per week (MET-min/W), calculated as the sum of the three activity domains multiplied by the respective estimated METs: walking/light (x 3.3 METs), moderate (x 4.0 METs) and vigorous activities (x 8.0 METs). For detailed information see the IPAQ Scoring Protocol, 2005, p. 5: [26]. According to Craig et al. [28], the reference value for adults independent of the sex is 2514 MET-min/W (median - MD). In addition, the reported times for moderate and vigorous PA are summed and expressed as MVPA-min/W. The PA-levels were categorized as sufficient or insufficient based on the PA-guidelines of the WHO [2].

#### Sitting time (ST)

The ST was analyzed by the IPAQ-SF. The question on the ST was developed as a separate indicator and is not part of the overall score for PA. The parents were asked to think about the total time in minutes per workday (min/WD) they spent sitting before and during their child’s cancer therapy.

### Outcome measures

#### Primary endpoint - PA

Primarily, this study aimed to compare the parents’ PA before diagnosis with the PA during their child’s cancer therapy, consisting of inpatient (Hospital) and outpatient (Home) treatment phases.

#### Secondary endpoints - ST

The extent to which parents’ ST is influenced during their child’s cancer therapy was examined.

The questionnaire comprises a third part in which we asked self-generated questions about the attitude towards exercise, the current physical status of the parents since the child’s cancer diagnosis. Parents’ physical complaints, their interest in (family-oriented) exercise programs and the necessary framework conditions from the parents’ point of view are determined.

### Statistical analyses

For the descriptive statistics and characterization of the parents and patients, we used percentages (%), arithmetic means, standard deviations (SD), median (MD), and ranges. The intraindividual comparisons of parental PA-behavior before diagnosis, during inpatient and outpatient stays with their child were analyzed using a one-factor analysis of variance (ANOVA) with repeated measures. All analyses were conducted using IBM SPSS Statistics (version 23.0). The level of statistical significance was set at *p* < 0.05. The analysis of the self-generated questions included frequencies (*n*) and percentages (%).

## Results

**Fig. 1:**
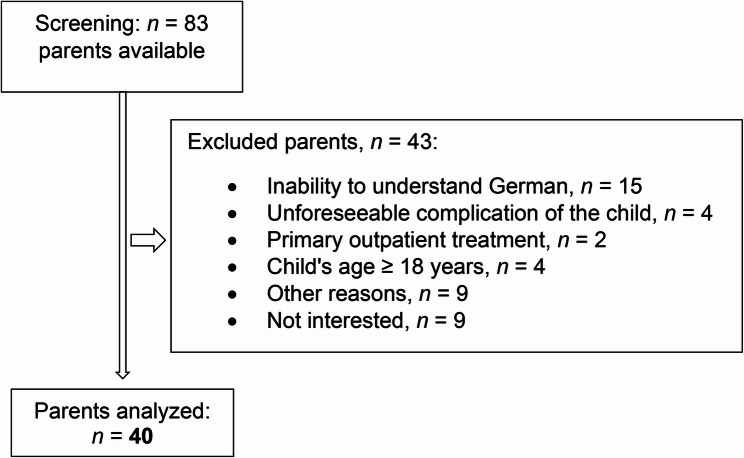
Flow diagram of screening and study recruitment

A total number (*n*) of 83 parents were available for the survey during the study period (Fig. 1). Out of 49 parents invited to participate, nine declined, resulting in a response rate of 81.6%. The analysis included 40 parents (female: *n* = 31 (77.5%), male: *n* = 9 (22.5%)) of 31 children. For nine children, both parents took part in the survey (Table S1). The mean age was 40.1 years (range: 29–62 years) and the reported mean BMI was 25 ± 5.5 kg/m² (Table 1).

### Study cohort

**Table 1:** Characteristics of the study cohort (*n* = 40 parents). Results in *n* (%), mean ± SD and range

Characteristics of the parents (*n* = 40)	*n* (%)	mean ± SD; range
Age (years)		40.1 ± 8.2; 29–62
Body Mass Index (BMI; kg/m²):		25 ± 5.5; 18.9–42.2
*overweight (BMI 25–29.9 kg/m²)*	*13* (32.5)	
*obesity (BMI ≥ 30 kg/m²)*	*6* (15)	
Gender:		
*Male*	*9* (22.5)	
*Female*	*31* (77.5)	

### Reported PA of parents before and during their child’s cancer therapy

**Fig. 2:**
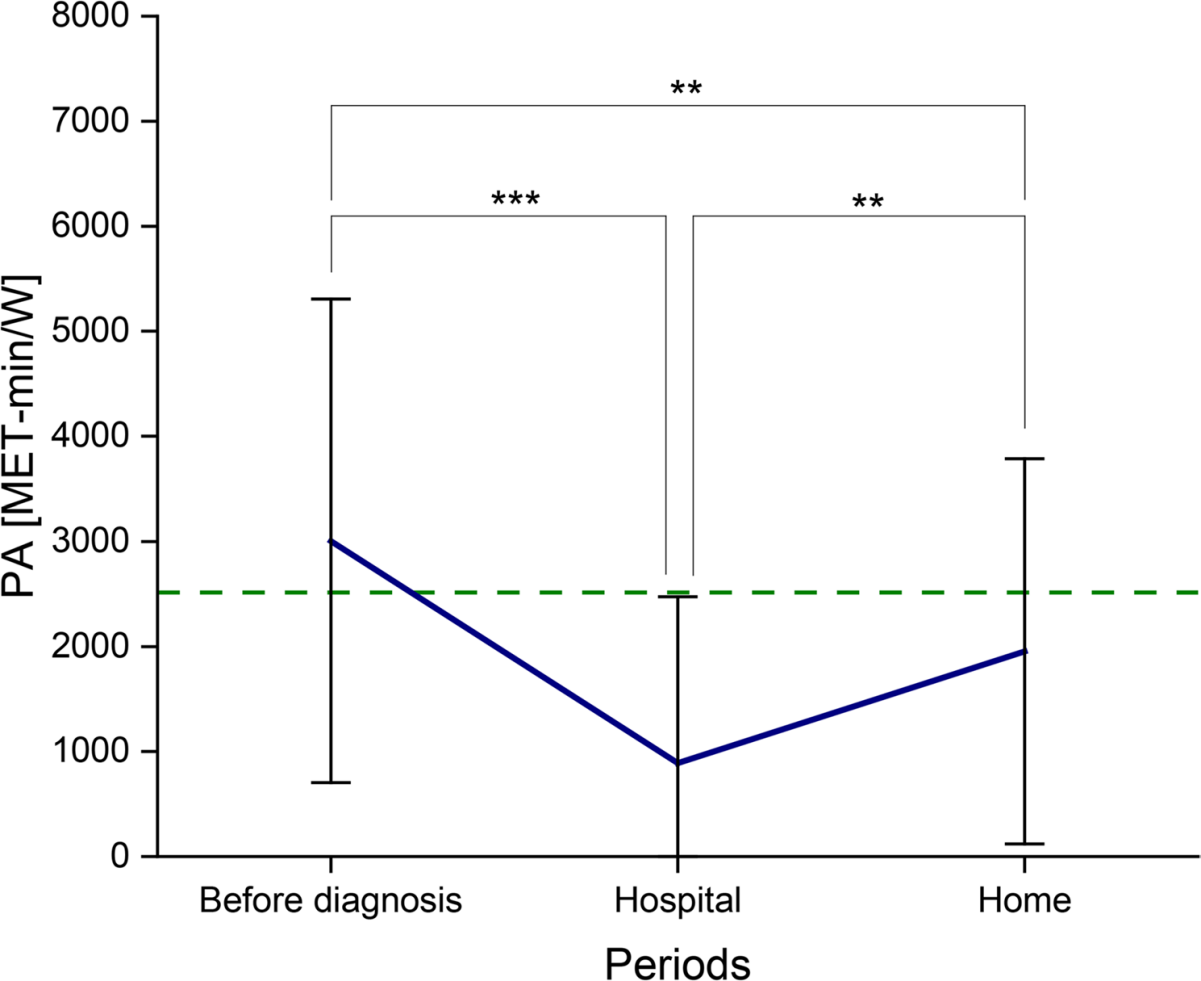
PA in MET-min/W of parents of children suffering from cancer (*n* = 40; mean ± SD) in the periods before diagnosis, during inpatient (Hospital) and outpatient stays (Home); the green dashed line represents the reference value as MD (2514 MET-min/W) according to Craig et al. [28]; MD before diagnosis in this study: 2592 MET-min/W; corresponding *p*-values (^**^: *p* < 0.01; ^***^: *p* < 0.001)

Before the cancer diagnosis of the child, parental self-reported PA (mean ± SD in MET-min/W: 3004.5 ± 2301.4; MD: 2592) corresponded with the reference [28]. Reported parental PA differed significantly during inpatient stays with their child compared to the time before the diagnosis. The stated PA values decreased during inpatient stays (Hospital: 889.1 ± 1585.1; MD: 231; *p* < 0.001). Even during outpatient stays, PA significantly decreased compared to that before diagnosis (Home: 1953.3 ± 1832.7; MD: 1546; *p* < 0.01). Likewise, significant differences between inpatient and outpatient stays existed, with an increase in presented PA at home (*p* < 0.01) (Fig. 2).

### Comparison with PA-guidelines of the WHO

Before the child’s cancer diagnosis, 52.5% of parents fulfilled the WHO recommendations for PA (≥ 150 MVPA-min/W or ≥ 75 min/W of vigorous PA) and 47.5% did not meet the criteria (Table 2). The reported mean MVPA-value was 233.8 min/W (Table 3). During inpatient stays with their child, 95% of parents were insufficiently active with an average reported MVPA of 41.6 min/W. When parents were at home with their child, this percentage dropped to 75% not meeting PA-guidelines. A weekly self-reported MVPA of 126.6 min was achieved.

**Table 2:** Degree of compliance with the WHO PA-guidelines of parents of children suffering from cancer (*n* = 40; *n* (%)) in the periods before diagnosis, during inpatient (Hospital) and outpatient stays (Home)

WHO PA-guidelines	Before diagnosis	Hospital	Home
fulfilled (sufficiently active)	*21* (52.5)	*2* (5)	*10* (25)
not fulfilled (not sufficiently active)	*19* (47.5)	*38* (95)	*30* (75)

**Table 3:** MVPA in min/W of parents of children suffering from cancer (*n* = 40; mean ± SD and MD) in the periods before diagnosis, during inpatient (Hospital) and outpatient stays (Home)

MVPA in min/W	Before diagnosis	Hospital	Home
mean ± SD	233.8 ± 253.7	41.6 ± 144.1	126.6 ± 205.3
MD	172.5	0	45

### Reported ST of parents before and during their child’s cancer therapy

**Fig. 3:**
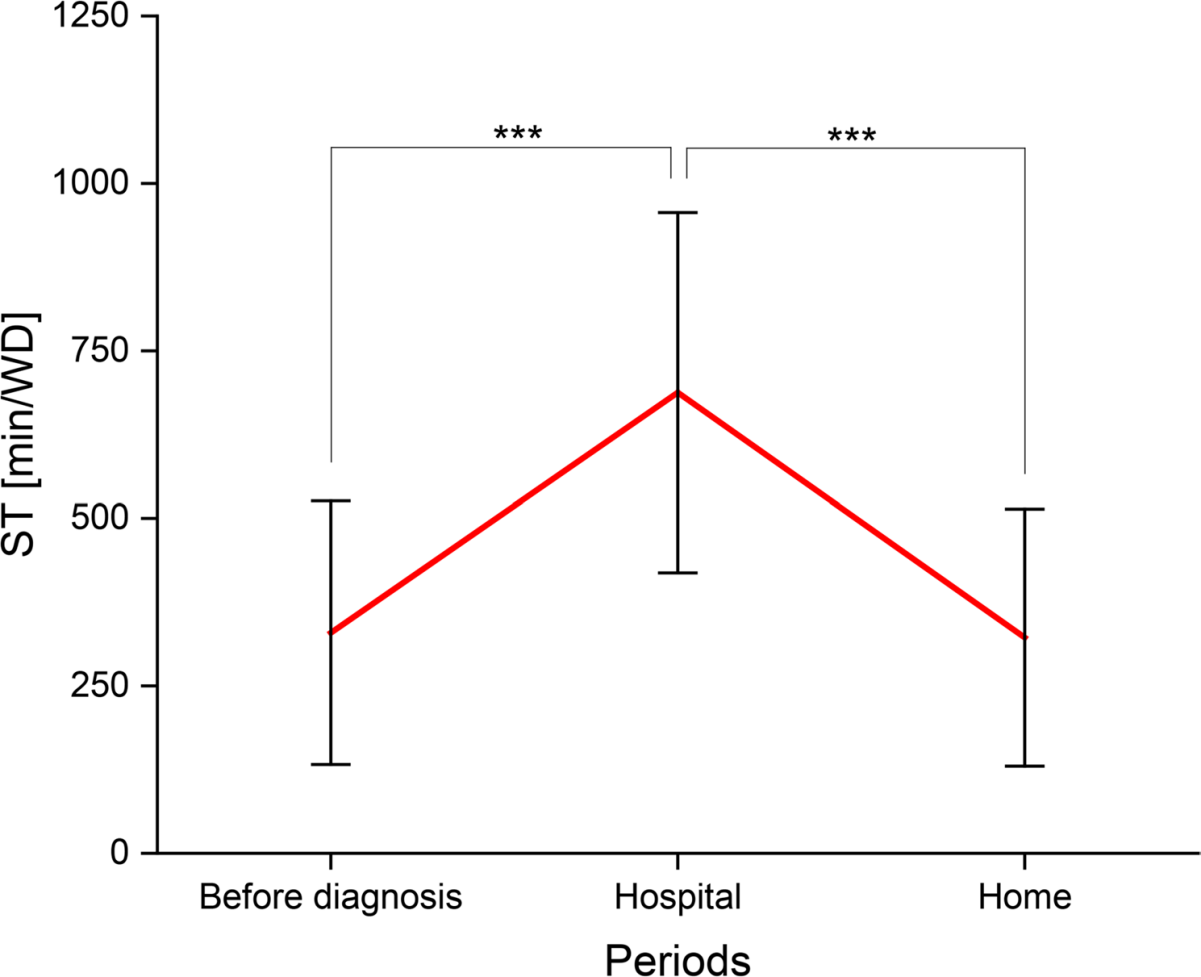
ST in min/WD of parents of children suffering from cancer (*n* = 40; mean ± SD) in the periods before diagnosis, during inpatient (Hospital) and outpatient stays (Home); corresponding *p*-value (^***^: *p* < 0.001)

The parents’ reported ST (mean ± SD in min/WD) demonstrated a significant difference in the periods before their child’s cancer diagnosis compared with inpatient stays (Before diagnosis vs. Hospital: 329.3 ± 196.6 vs. 687.8 ± 268.8; *p* < 0.001). The documented ST doubles to more than 10 h/WD (Hospital). At home, the stated ST decreased (Hospital vs. Home: 687.8 ± 268.8 vs. 322.1 ± 191.8; *p* < 0.001). The reported ST before diagnosis and during the outpatient period at home did not differ among the parents surveyed (Fig. 3).

Out of all parents, 45% (*n* = 18) reported from new physical complaints since their child’s diagnosis. Frequently mentioned complaints included tension, shoulder, head, and back pain, weight gain, and stomach pain. After providing certain conditions, 92.5% of the parents (*n* = 37) could imagine taking part in an exercise program (Table 4). Most of the parents preferred a separate room to exercise in the hospital (87.5%, *n* = 35).

**Table 4:** Self-reported physical complaints and interest in exercise programs of parents of children suffering from cancer (*n* = 40; *n* (%))

Parental information (*n* = 40)	*n* (%)
Physical complaints since the child’s cancer diagnosis	*18* (45)
Interested in a clinic-internal exercise program	*37* (92.5)
Sports/exercise room	*35* (87.5)

## Discussion

The present study examined the PA and ST of parents of children suffering from cancer before and during cancer therapy using a questionnaire. The self-reported, retrospective physical inactivity rate among parents prior to their child’s cancer diagnosis was similar to the WHO estimate for the general adult population in Germany (55% [8] vs. 47.5% (Table 2)), allowing for a certain degree of comparability.

Reported parental PA-levels decreased significantly throughout their child’s cancer therapy. The greatest reduction occurred during inpatient stays (Fig. 2). During this time, parents’ reported PA was far from the reference value according to Craig et al. [28], and nearly none of the parents met the guidelines of the WHO (Table 2). Compared with available data on the German adult population [8], we demonstrated a greater degree of noncompliance with PA-recommendations during inpatient stays (55% [8] vs. 95% (Table 2)).

The reason for the diminished level of self-reported PA can be manifold. First, the child’s state of health will influence the parents’ level and type of activity. The priorities of daily living change. Second, the lack of alternative places to stay, move, and rest and the resulting habit of performing all activities of daily living, such as eating, playing, and watching TV, at the child’s bedside will change the PA-level. The constant restriction due to the infusion stand and the resulting limited mobility of the child, exhaustion caused by insomnia [29], lack of time and (social) isolation to protect their child are several other reasons to take into consideration.

As PA decreased, the reported amount of time parents spent sitting during inpatient stays increased significantly. Assuming eight hours of sleep, the parents’ documented ST accounted for at least half of the total waking time. Compared to the time before cancer diagnosis, reported ST doubled to more than 10 h/WD (Fig. 3). A closer look at the parents’ ST during hospital stays revealed that 85% of the parents spent more than eight hours/WD and 62.5% even 10 h/WD sitting. Excessive sitting of more than eight hours per day has been linked to an increased risk of chronic diseases [9–11, 30], and is positively associated with the risk of premature mortality [31]. Based on current studies, our study cohort would need to engage in at least 60 min per day of MVPA to avert adverse health effects due to sitting [30, 32].

In this context, the extent of hospitalization provides important background information, as it further limits opportunities for PA. During the survey period (09/2021 to 02/2022), children of participating parents had an average of 45 hospital days (range: 5–85) (Table S1). While this reflects only the study period, it provides a rough estimate. Some children were already in later treatment phases and therefore had fewer hospital days compared to newly diagnosed patients. In Germany, children with cancer typically spend about 50% of their treatment time in hospital, including admissions due to treatment-related complications. Length and frequency of stays vary widely depending on diagnosis and treatment phase, which should be considered when evaluating activity levels and designing interventions.

In our cohort, stated PA increased slightly again during outpatient stays at home with the child, but did not reach the amount of reported PA at the time before diagnosis. In particular, parents’ observed walking times at home (mean: 543.6 min/W) are estimated to be longer than those during inpatient stays (mean: 206.2 min/W). This result is consistent with the findings of Winter et al. [13]: according to objective measures of step count, children with cancer have a higher activity level during outpatient compared to inpatient stays. Similarly, an increased number of the parents studied reached again the PA-recommendations of the WHO at home between hospital stays (Table 2). Like the parental reported PA, the documented ST changed again from inpatient to outpatient stays and decreased significantly (Fig. 3). A compensation of the low amount of PA and the high amount of ST during inpatient stays, by e.g. increased walking in the period at home, could explain these findings. In this context, especially hospitalization with the child affected by cancer seems to be a specific risk factor for inactivity. Therefore, the time out of hospital should compensate for the losses of the family’s PA.

Furthermore, almost half of the parents reported from new physical complaints since their child’s cancer diagnosis (Table 4). These results were in line with the data from Wiener et al. [33]. Their results showed a deterioration in the general health of 40% of parents 6 to 18 months after their child’s cancer diagnosis. This deterioration included poorer dietary habits, reduced PA and less time for pleasurable activities. Moreover, parents of children with cancer reported significantly lower health-related quality of life [21] due to increased caregiving responsibilities and numerous stressors, such as insomnia, financial problems, role conflict, disruption of daily routines, and uncertainty about the child’s prognosis [32]. In addition, many parents suffered from anxiety and depression years after their child’s diagnosis [34, 35]. Moreover, 32.5% of our cohort already reported from overweight (Table 1).

Taken together, a considerable proportion of parents seems to put their own health at risk during their child’s illness due to insufficient PA and prolonged ST. Since a child’s cancer therapy may last for months or even years, additional health issues due to inactivity may develop, but were not evaluated in this study. As parents form an important support system for their ill child, they are expected to remain functional despite considerable psychological strain and health-related risk factors [1, 36]. It can be assumed that many parents experience not only psychological stress but also physical underload. This is supported by the fact that most respondents could genereally imagine participating in exercise programs, given appropriate conditions such as access to a dedicated exercise space (Table 4).

Finally, the results necessitate an intensification and consolidation of family-oriented movement offerings during childhood cancer therapy. Clinics should be designed to be more active (e.g. with a sports program and a sports room as a place to retreat and relax) in order to create more incentives to exercise with the aim of leaving the hospital bed or room more often. Preventive interventions including a combination of knowledge transfer and supervised family-oriented exercise programs in pediatric cancer wards, are necessary to reduce the time spent sitting and increase the PA of affected families. Such programs bear the chance to reduce inactivity-related and other long-term side effects of childhood cancer patients and their families.

This study has several limitations. First, retrospective surveys always carry the risk of bias due to subject recall with regard to the assessment of reported PA and ST during different periods, when the survey is carried out at only one time point after start of cancer therapy [37]. Second, the use of self-reports to assess PA and ST is another limitation. Self-reports has been proven to be less valid than objective measurements, such as accelerometry [38]. Nevertheless, this form of questionnaire was the only way to capture parents’ past activities and identify intraindividual changes. The questionnaire is used in nationwide epidemiological surveys and offers flexibility in terms of timeframes. In addition, the third part of the questionnaire on attitudes, the parents’ current situation since the child’s illness and prerequisites for movement therapy was developed and used for the first time for this study. It should be noted that these are self-generated questions and have not been tested for quality criteria. For future use, testing for objectivity, reliability and validity may be considered. Last, having performed a monocentric study, the results cannot be generalized to the entire population of parents of children suffering from cancer. To increase data quality and validity, a multicenter approach with a larger number of subjects will be necessary. This would not only enhance statistical power but also allow for the identification of meaningful clusters or subgroups, ultimately enabling the development of clearer, more targeted recommendations for supporting affected families.

## Conclusions

The results of this cross-sectional study indicate that parents experience a significant decrease in reported PA during their child’s cancer therapy. The greatest decrease in stated PA occurs during inpatient stays, with a corresponding significant increase in reported ST. Since almost half of the parents reported new physical complaints since their child’s cancer diagnosis and reported to be overweight, there seems to be a need to counteract these health hazards. Almost all parents can imagine participating in an internal exercise program at the clinic under certain conditions (e.g. in a sports/exercise room). Appreciating that parental PA can significantly affect their child’s behavior during and after completion of cancer therapy, future supervised exercise programs in pediatric oncology should include parents to reduce inactivity-related long-term side effects of the whole family.

## Supplementary Information

Supplementary Material 1.

Supplementary Material 2.

Supplementary Material 3.

Supplementary Material 4.

## Data Availability

This article only includes summarized data of this study. The raw data is available from the corresponding author upon reasonable request.

## Abbreviations

ANOVA: Analysis of variance
BMI: Body Mass Index
IPAQ-SF: International Physical Activity Questionnaire-Short Form
MD: Median
MET: Metabolic Equivalent of Task
Min: Minute
MVPA: Moderate to vigorous physical activity
n: Number
PA: Physical activity
SD: Standard deviation
ST: Sitting time
W: Week
WD: Workday
WHO: World Health Organization
