# Supplementary material for: Assessing the physical activity of parents of children suffering from cancer: a cross-sectional study
Source: BMC Public Health. 2025 Nov 17;25:3969. doi: 10.1186/s12889-025-25455-5 (PMC12621409; doi:10.1186/s12889-025-25455-5)
Supplement: Supplementary file 1 — Supplementary Material 1. [file 12889_2025_25455_MOESM1_ESM.docx]

**Additional files**

**Additional file 1:**

**Table S1** Characteristics of children with cancer (n = 31; for 9 children both parents took part in the study). Results in n (%), mean ± SD and range

| Childrens' characteristic **(*n* = 31)** | ***n* (%)** | **mean ± SD; range** |
| --- | --- | --- |
| Age (years) |  | 7.8 ± 4.9; 1-17 |
| Gender: |  |  |
| *Male* | *18* (58.1) |  |
| *Female* | *13* (41.9) |  |
| Months since diagnosis |  | 8.2 ± 8.4; 1-34 |
| Days of hospitalization of the child 09/21-02/22 |  | 45.2 ± 17.8; 5-85 |
| Cancer type: |  |  |
| *Leukemia* | *13* (41.9) |  |
| *Lymphoma* | *2* (6.5) |  |
| *Solid tumor* | *11* (35.5) |  |
| *Brain tumor* | *5* (16.1) |  |
