# Supplementary material for: Assessing the physical activity of parents of children suffering from cancer: a cross-sectional study
Source: BMC Public Health. 2025 Nov 17;25:3969. doi: 10.1186/s12889-025-25455-5 (PMC12621409; doi:10.1186/s12889-025-25455-5)

**Appendix:**

**Ethics vote letter:**
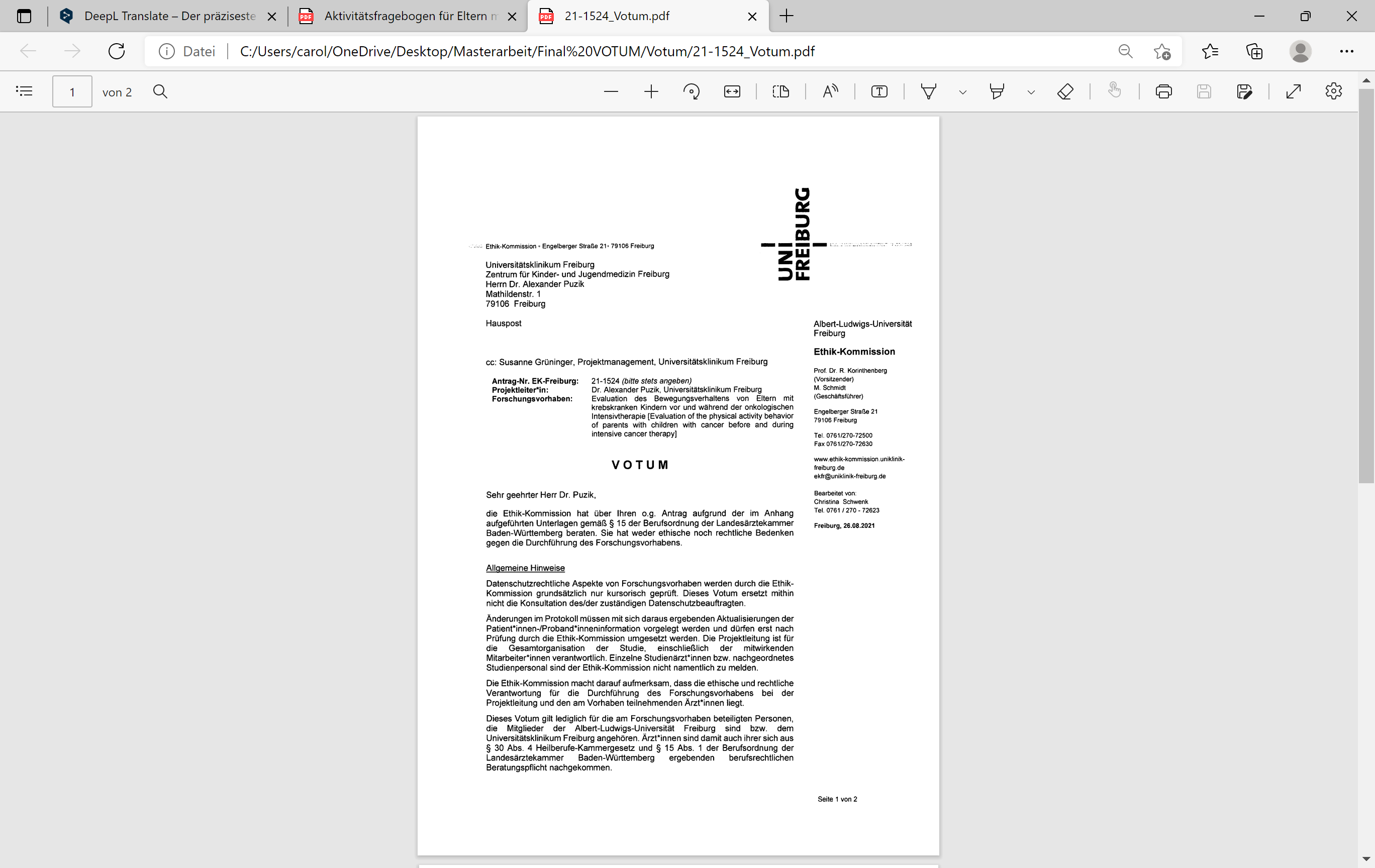


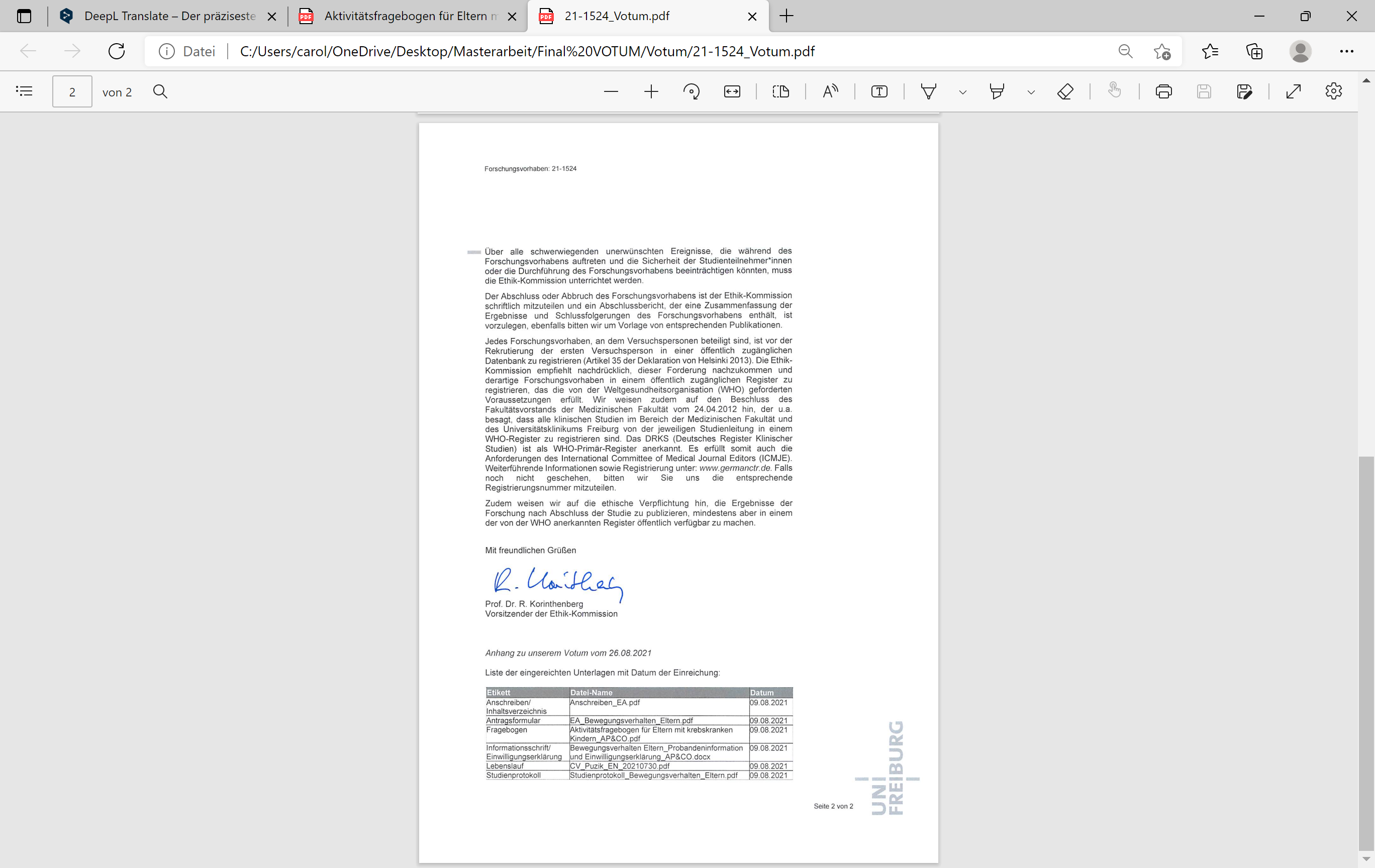


**Participant Information & Consent Form:**

German version (submitted and reviewed by the Ethics Committee):


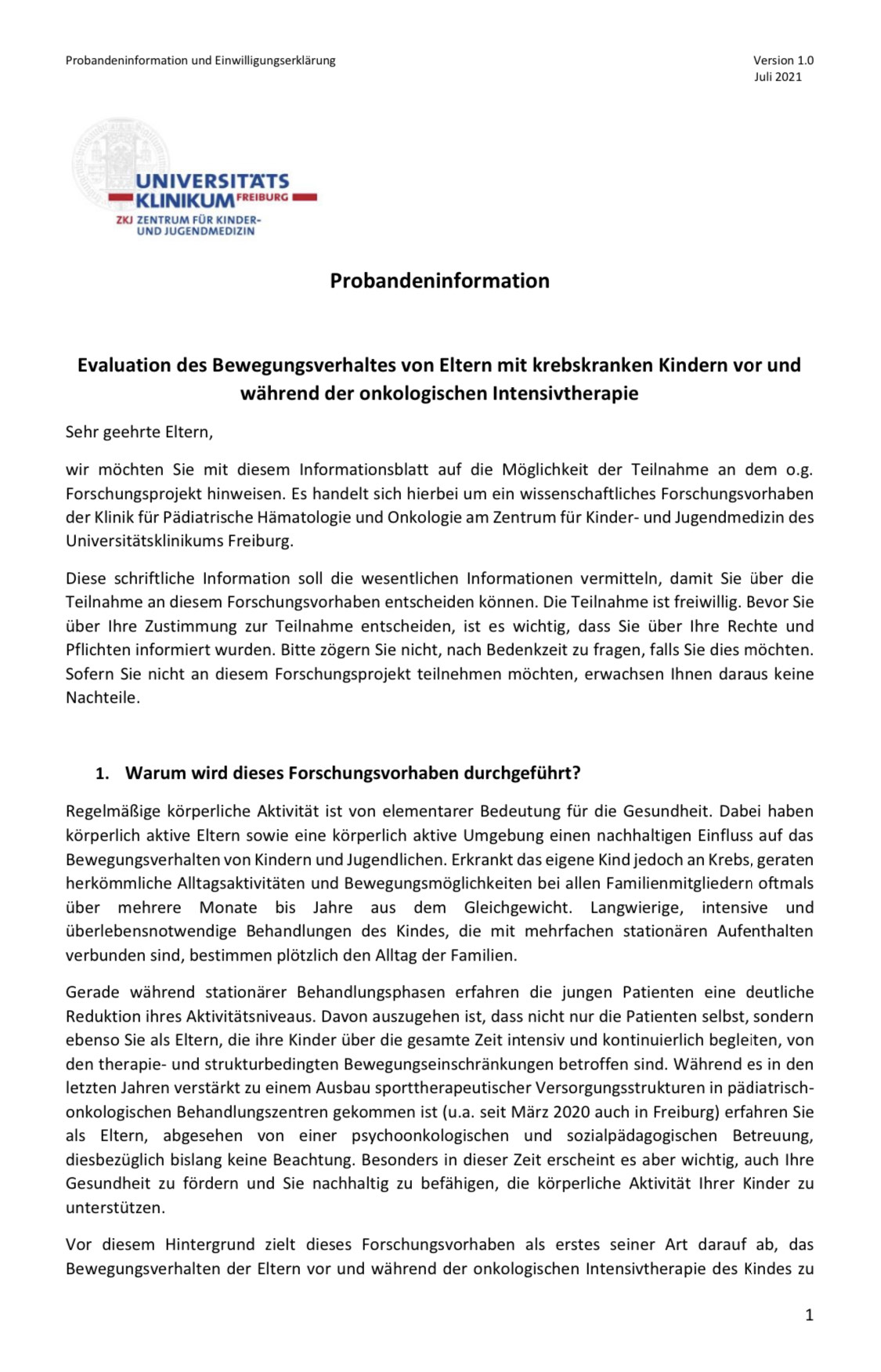


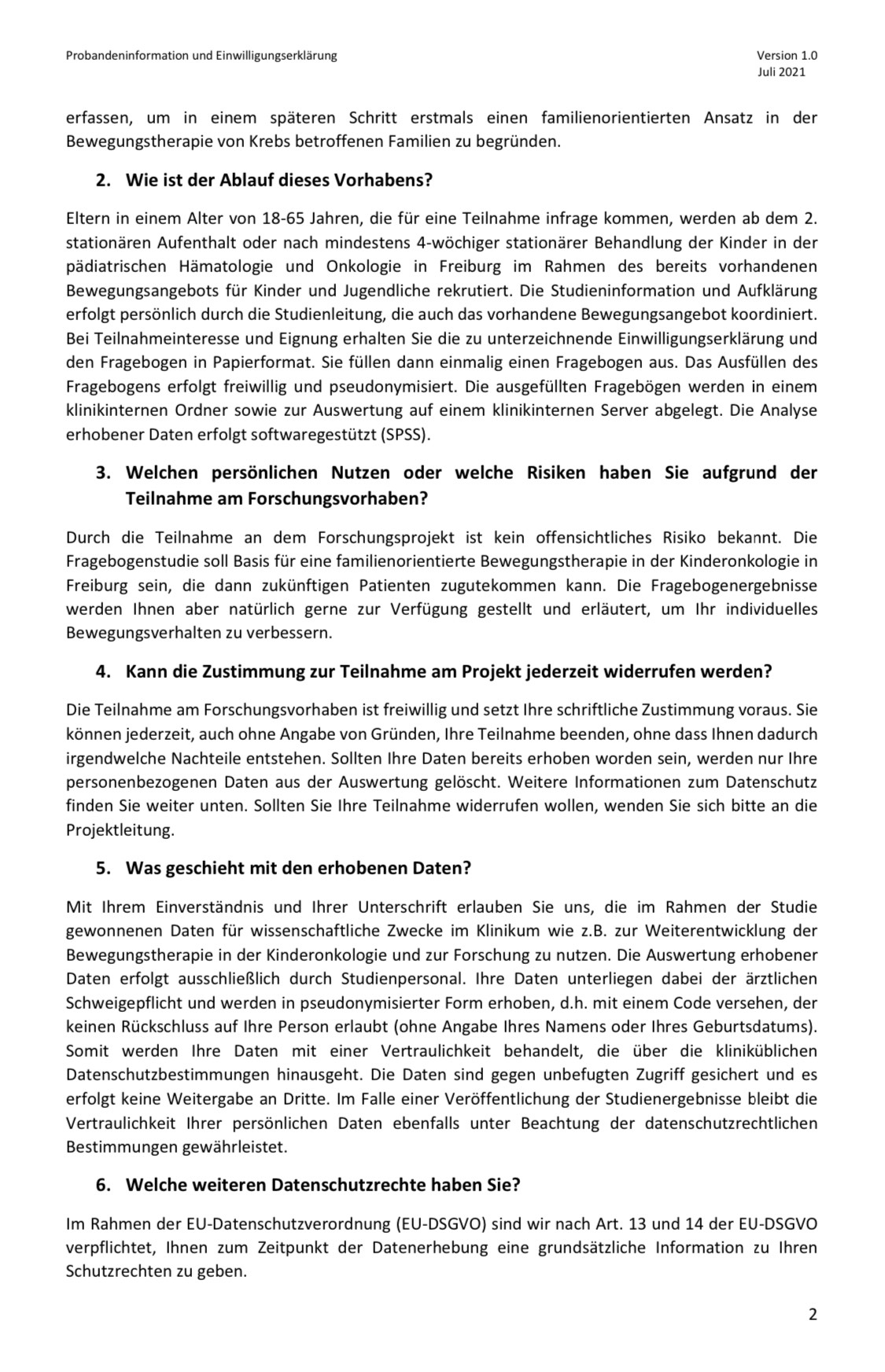

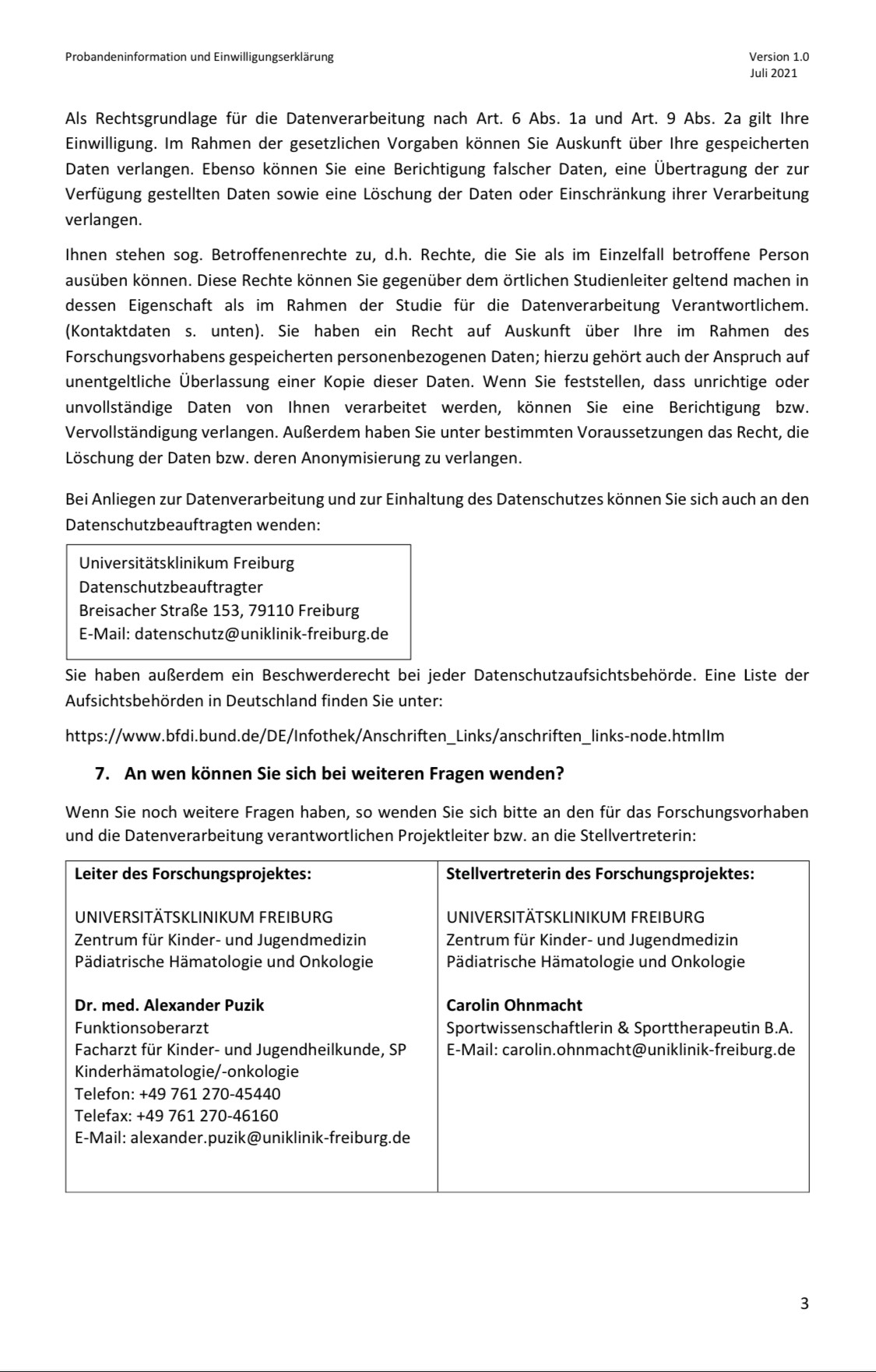


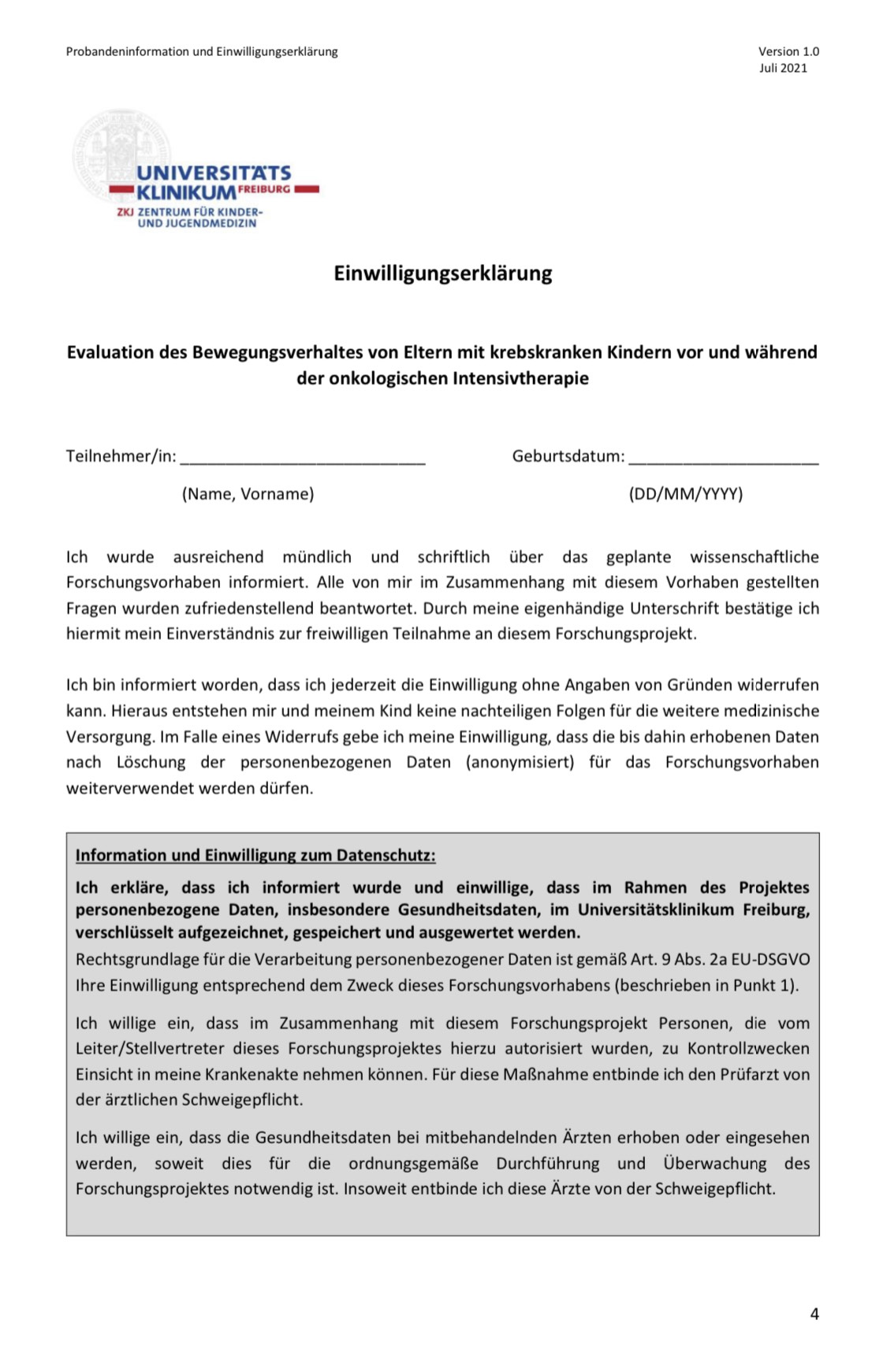


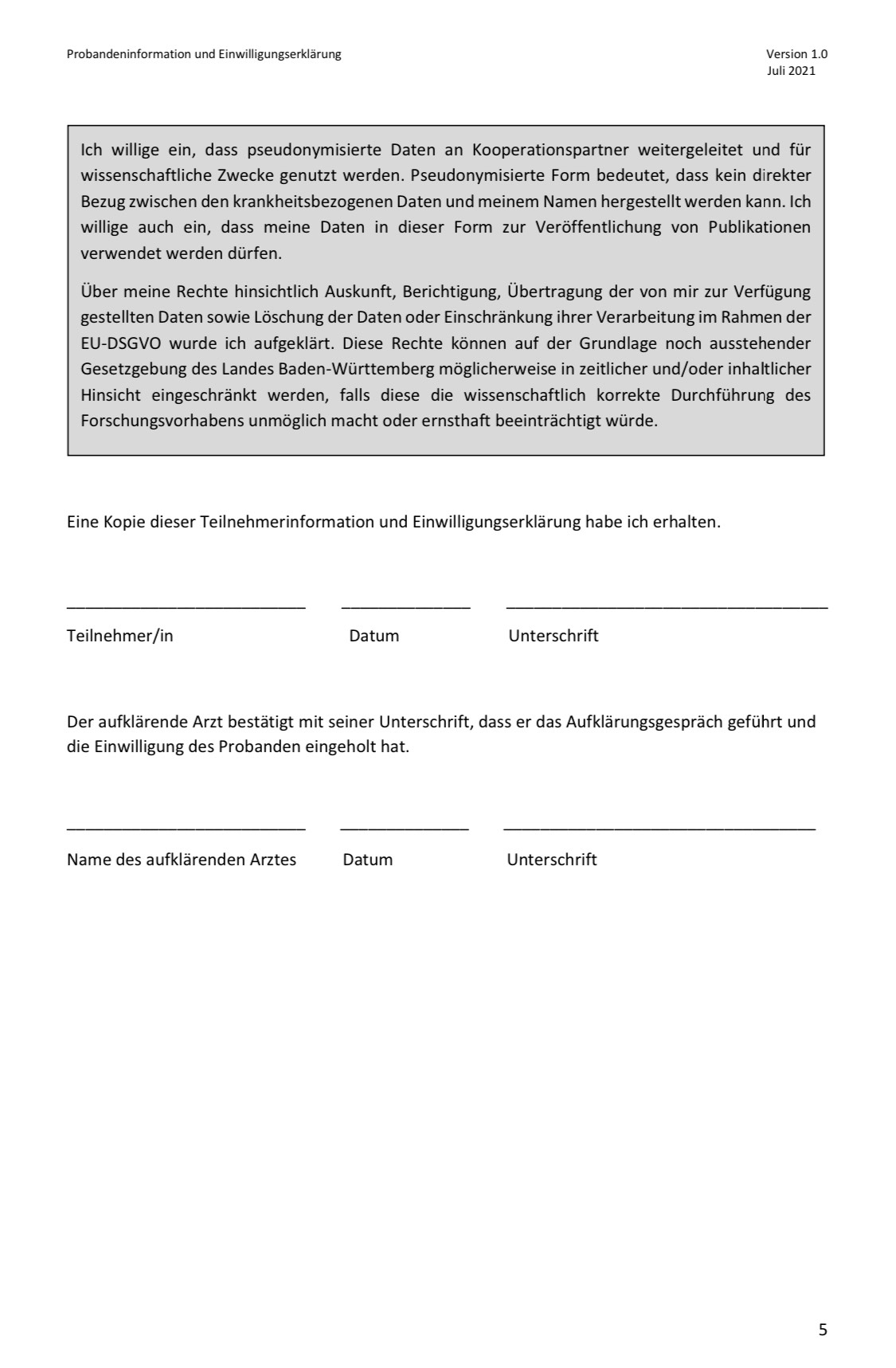


**Participant Information & Consent Form:**

English version (not submitted and not reviewed by the Ethics Committee 🡪 no original document, just for review):


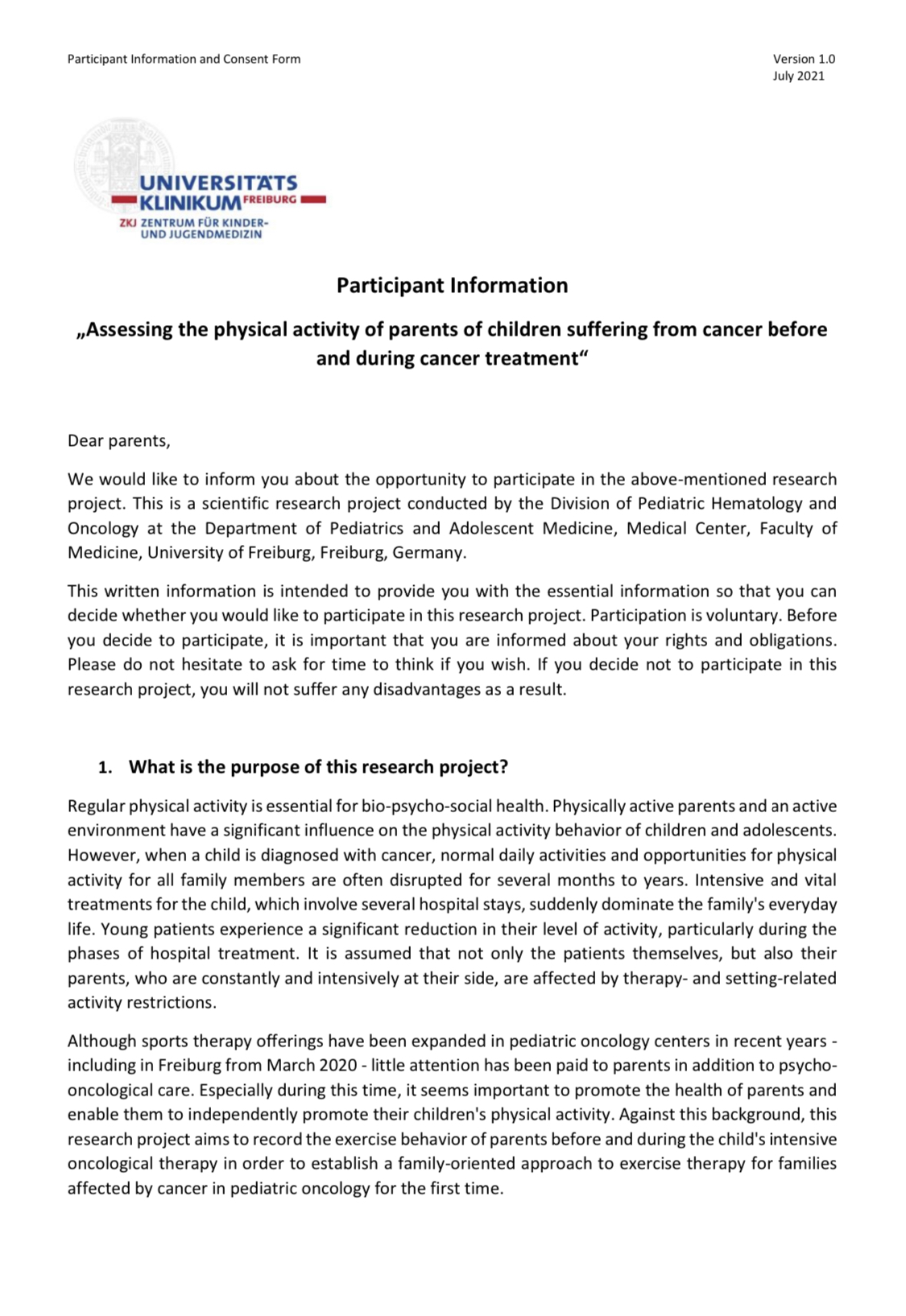


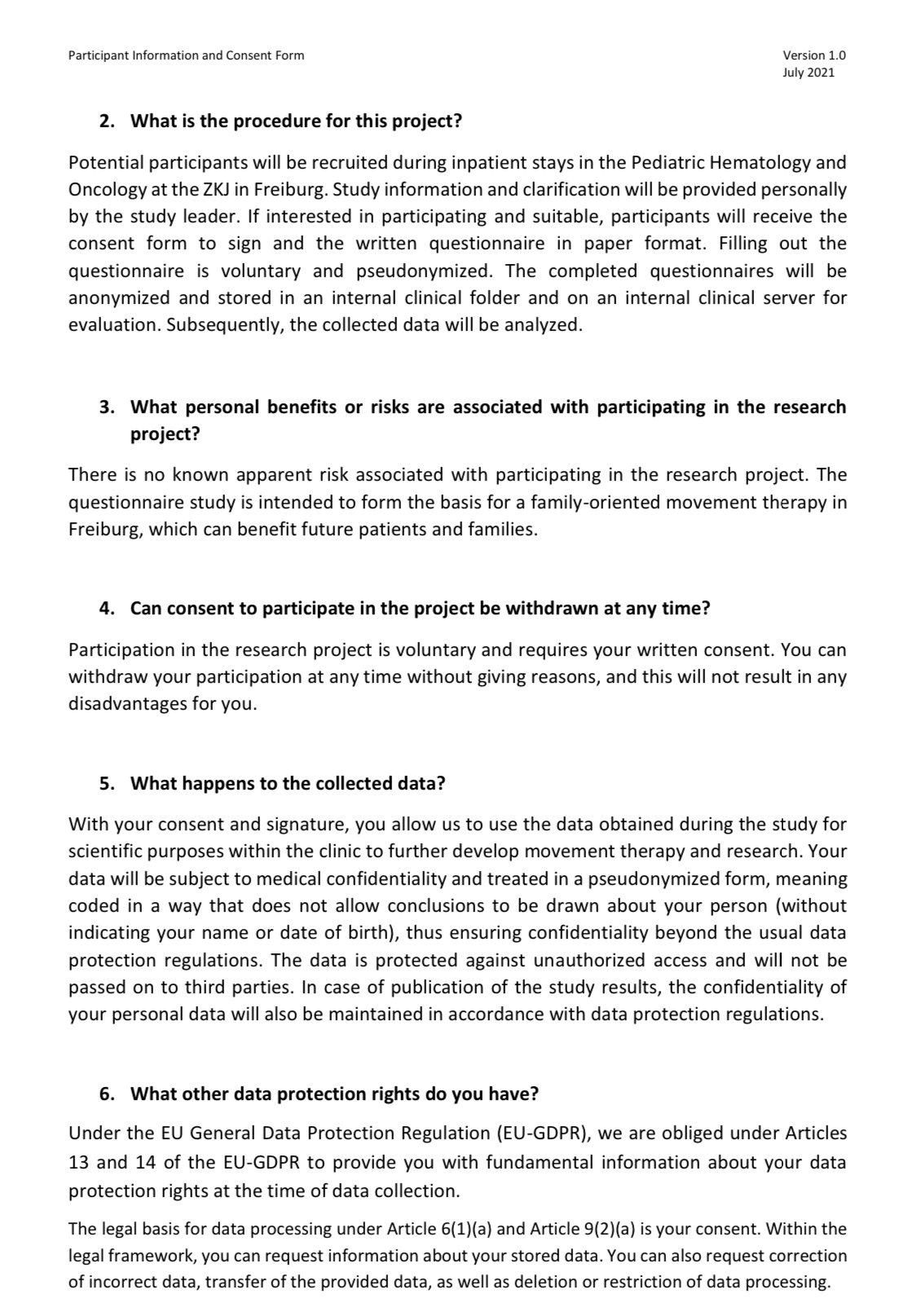


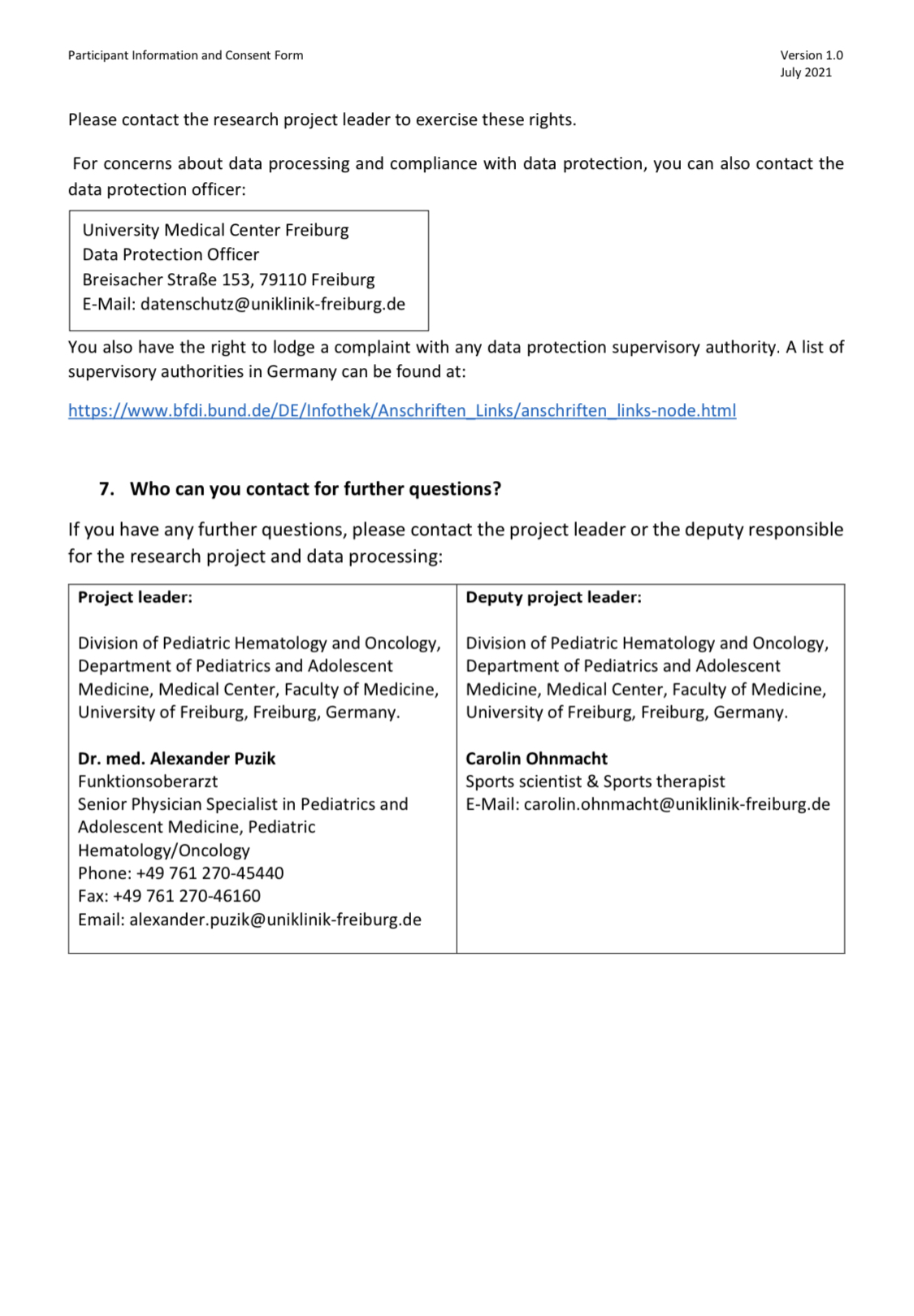


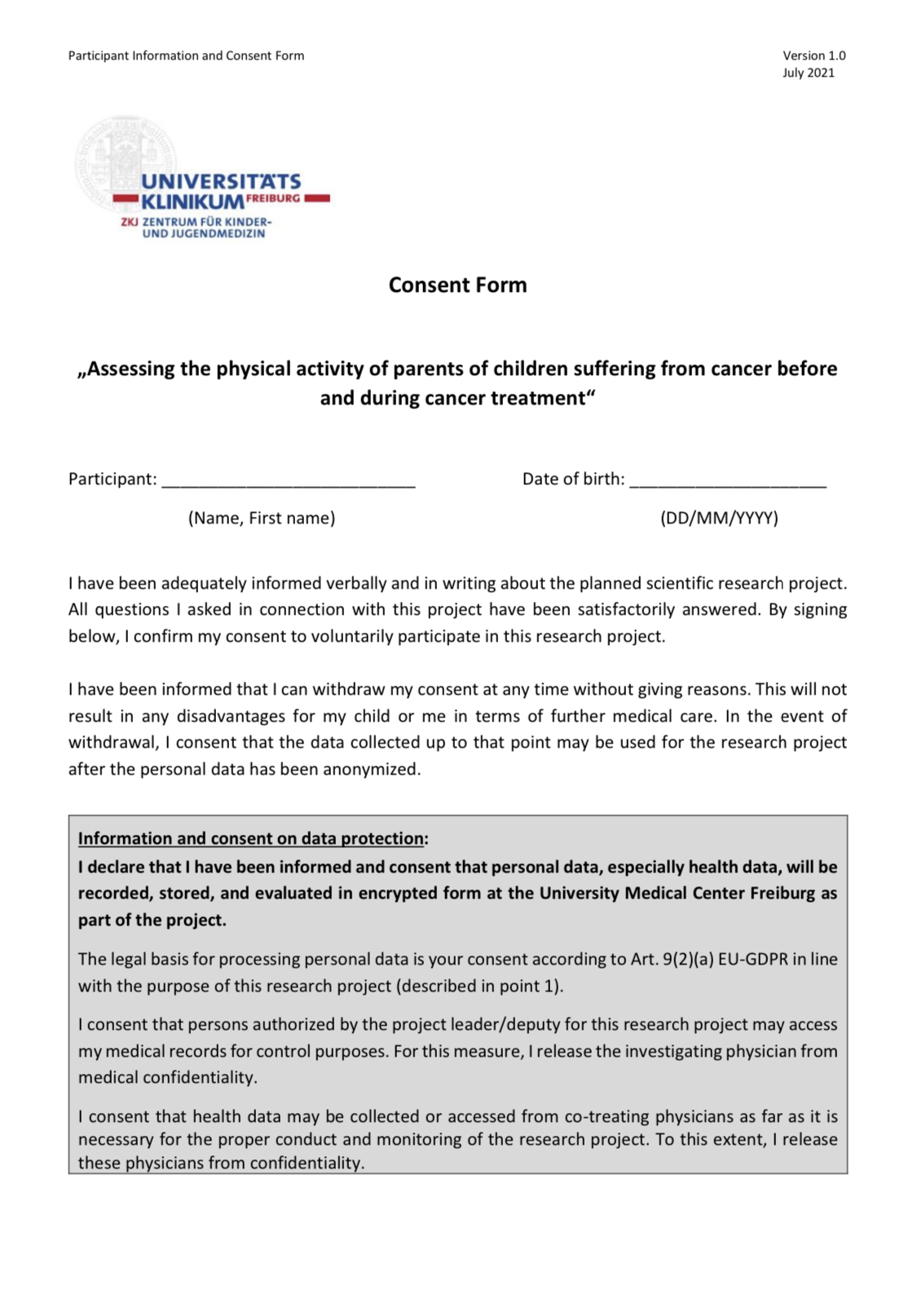


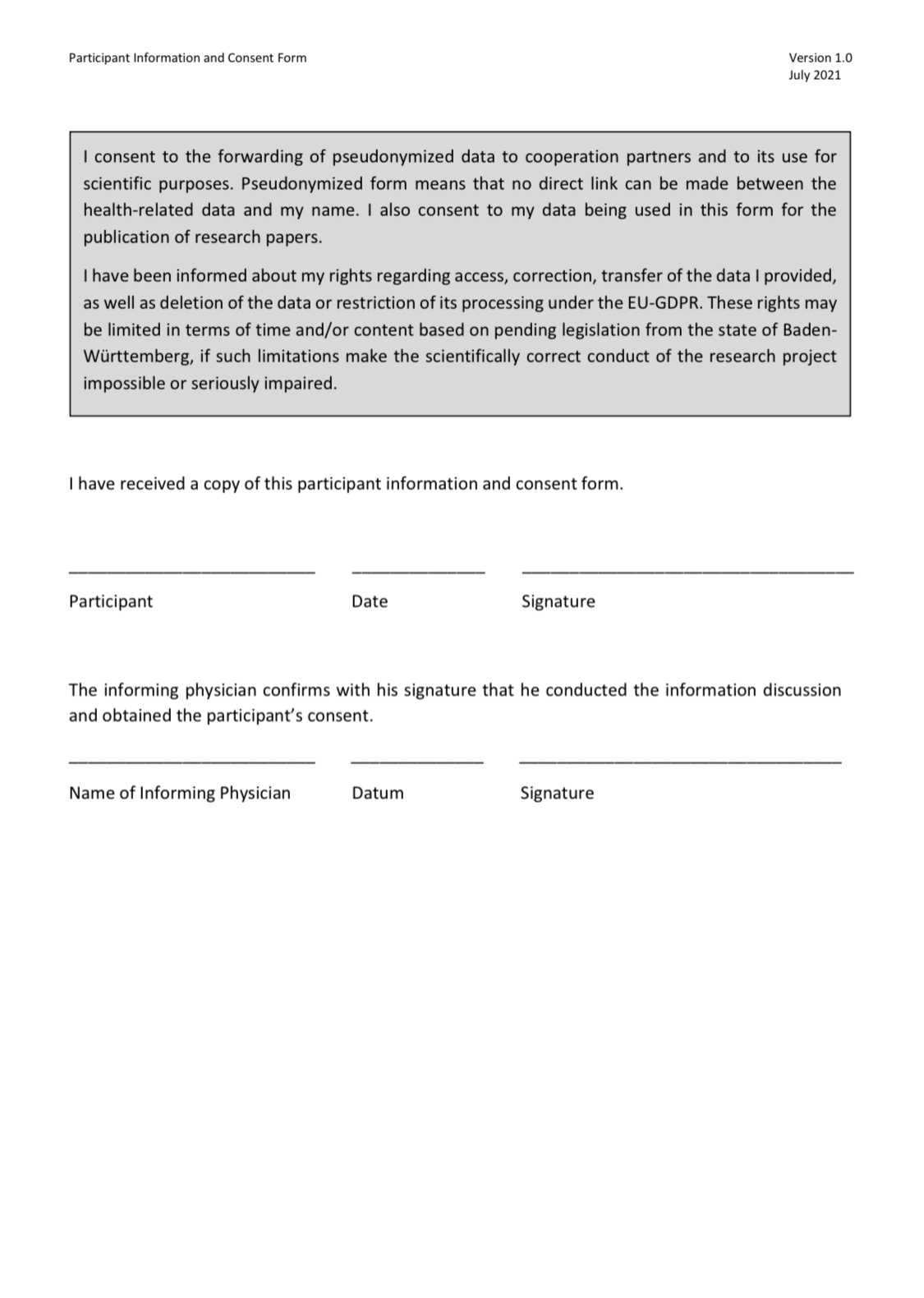


**Activity-Questionnaire:**

German version (submitted and reviewed by the Ethics Committee):


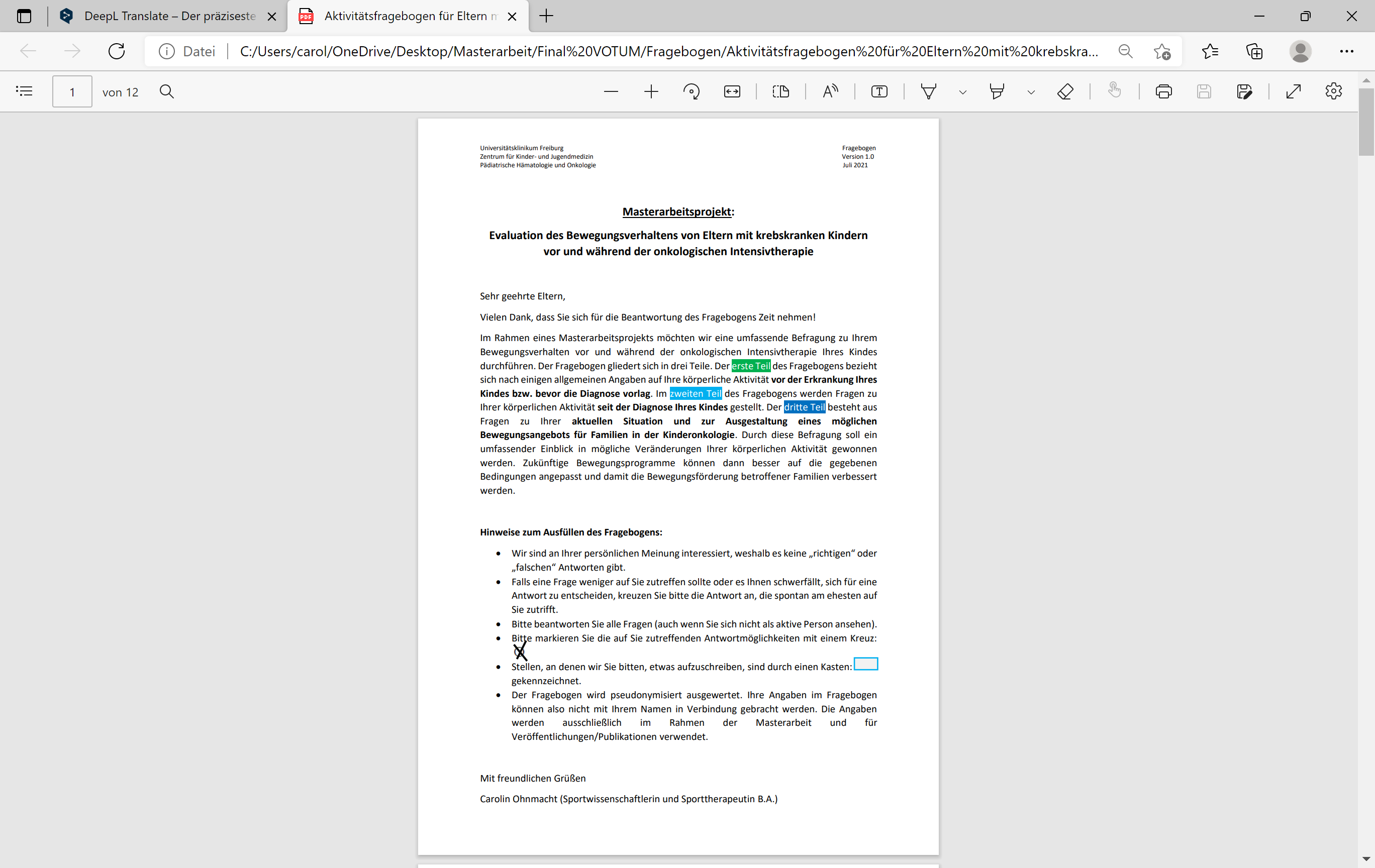


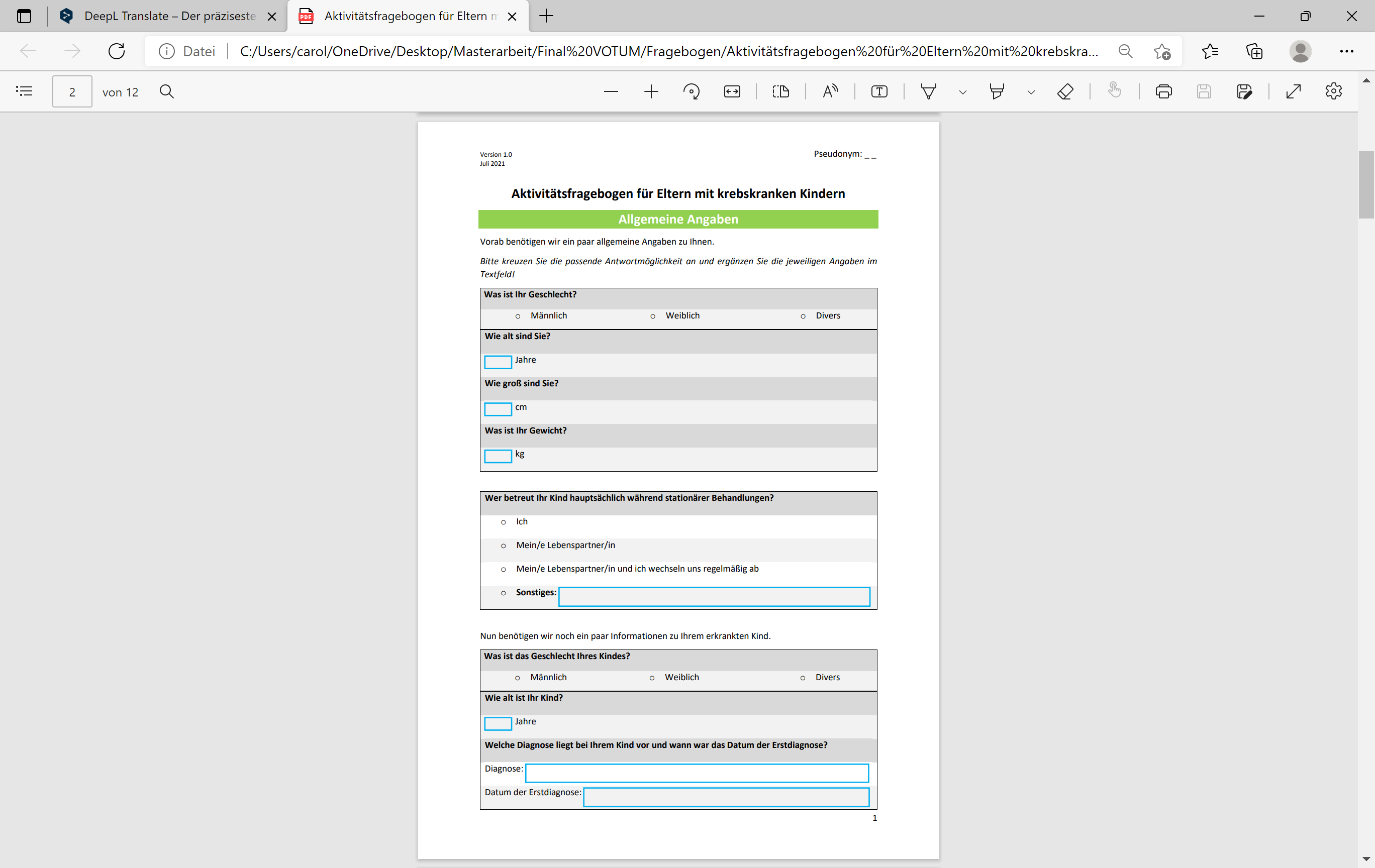


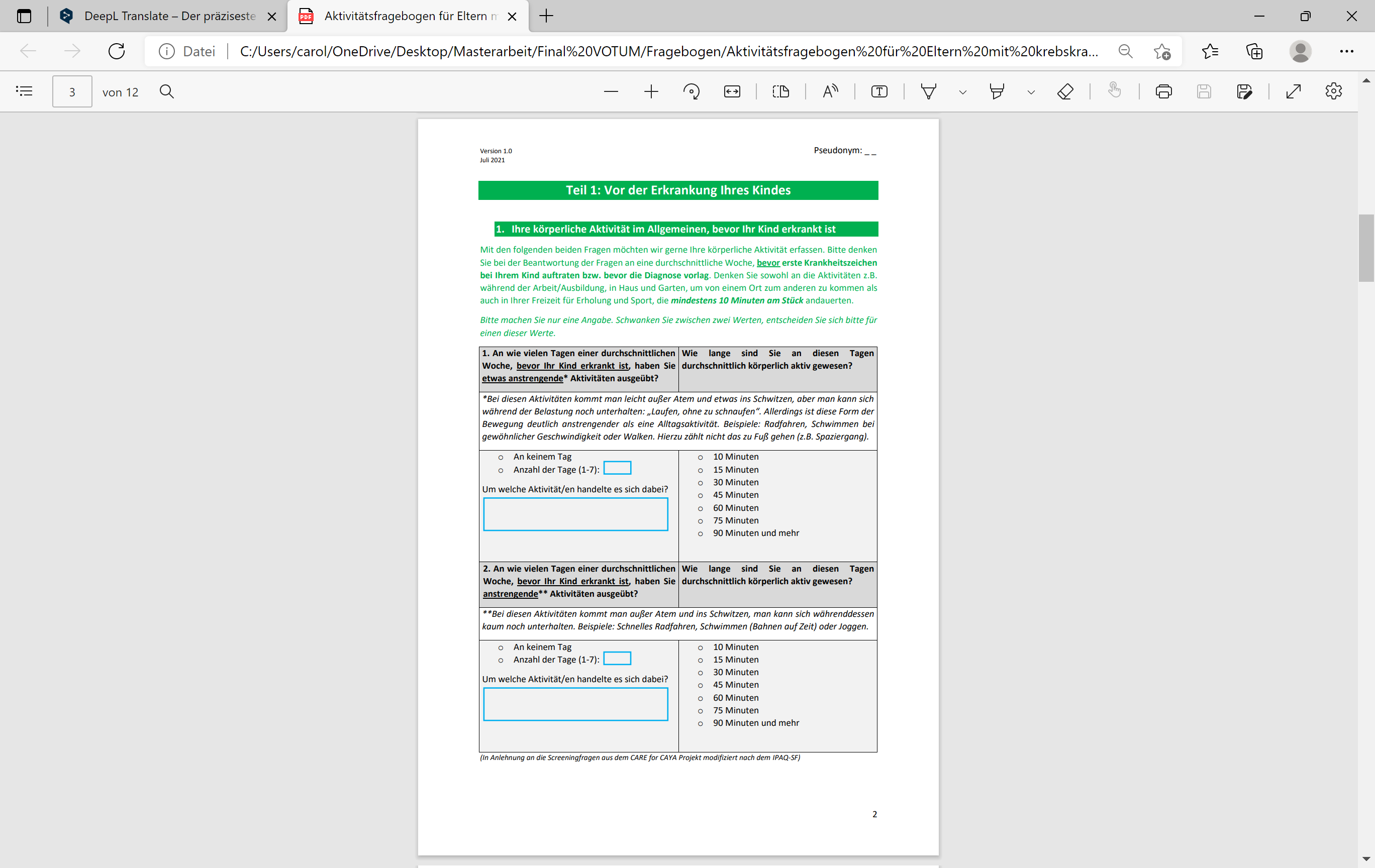


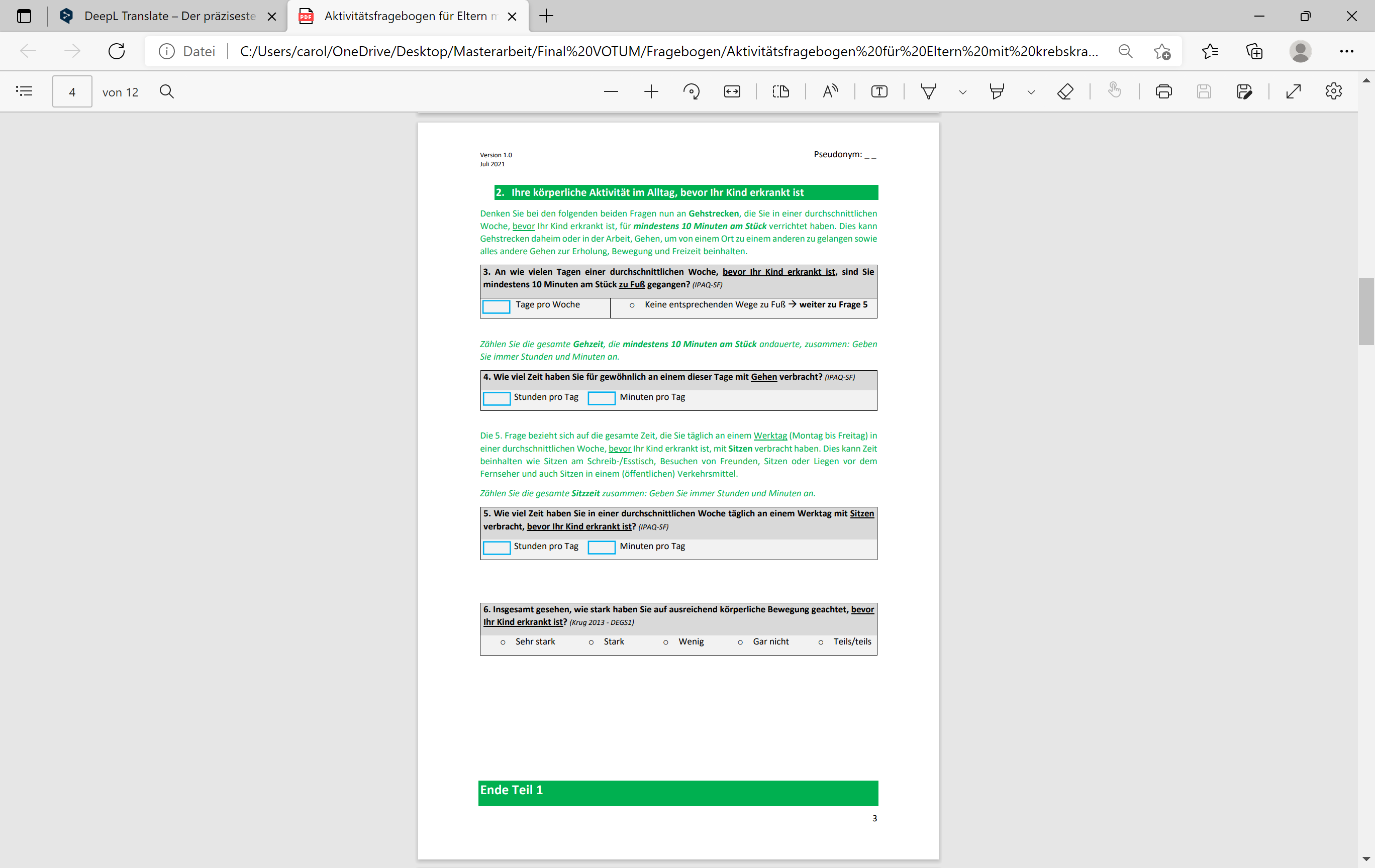


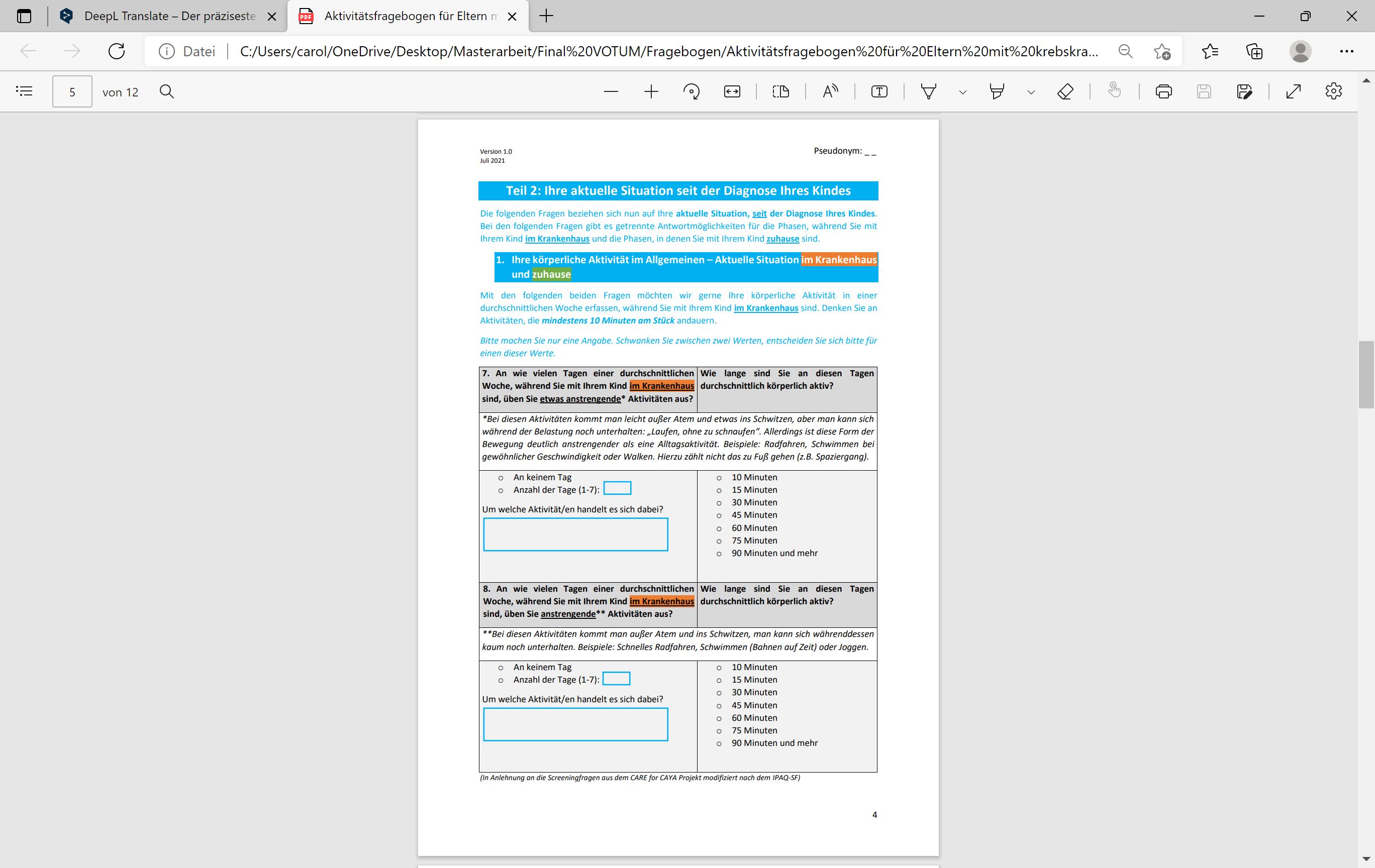


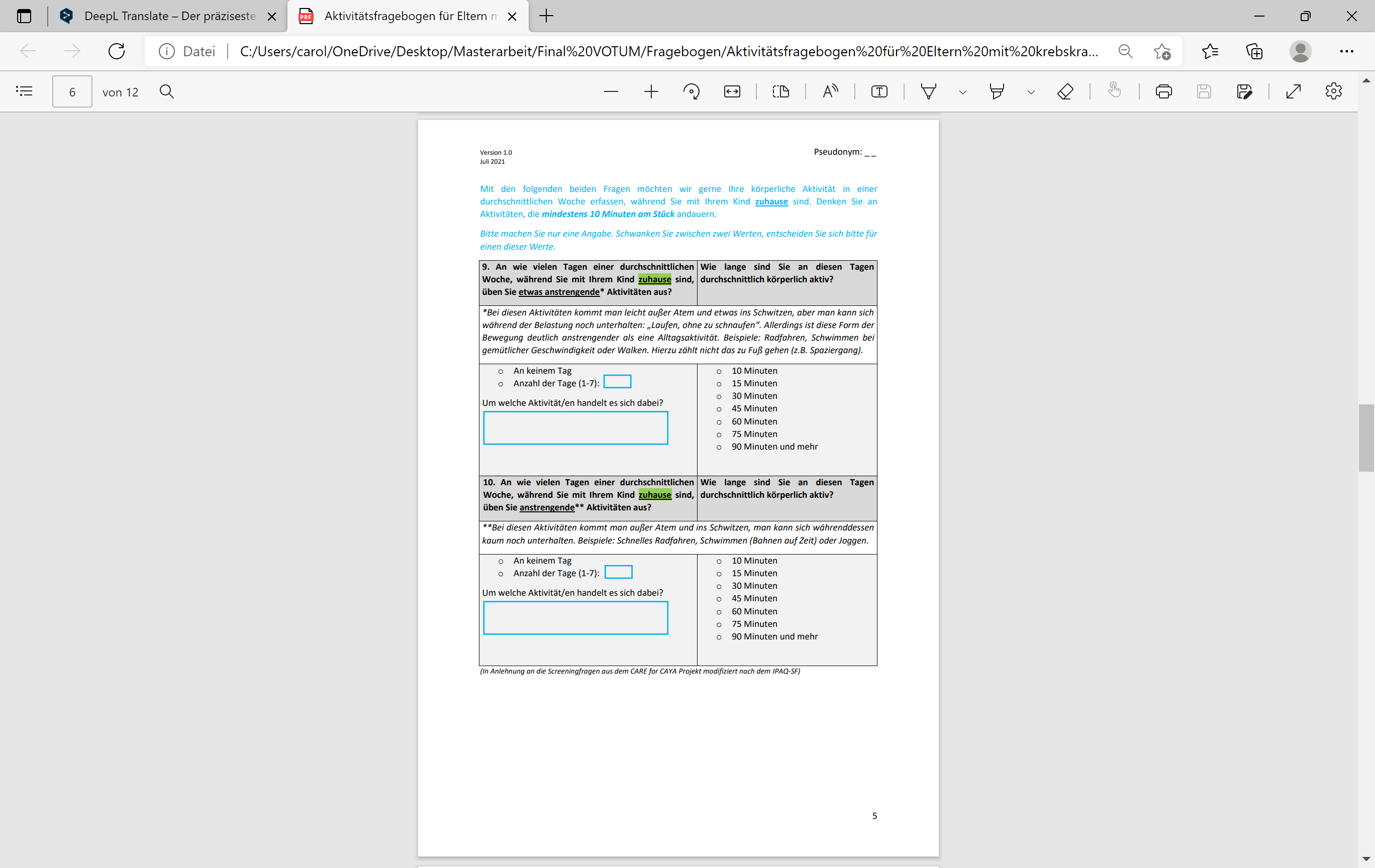


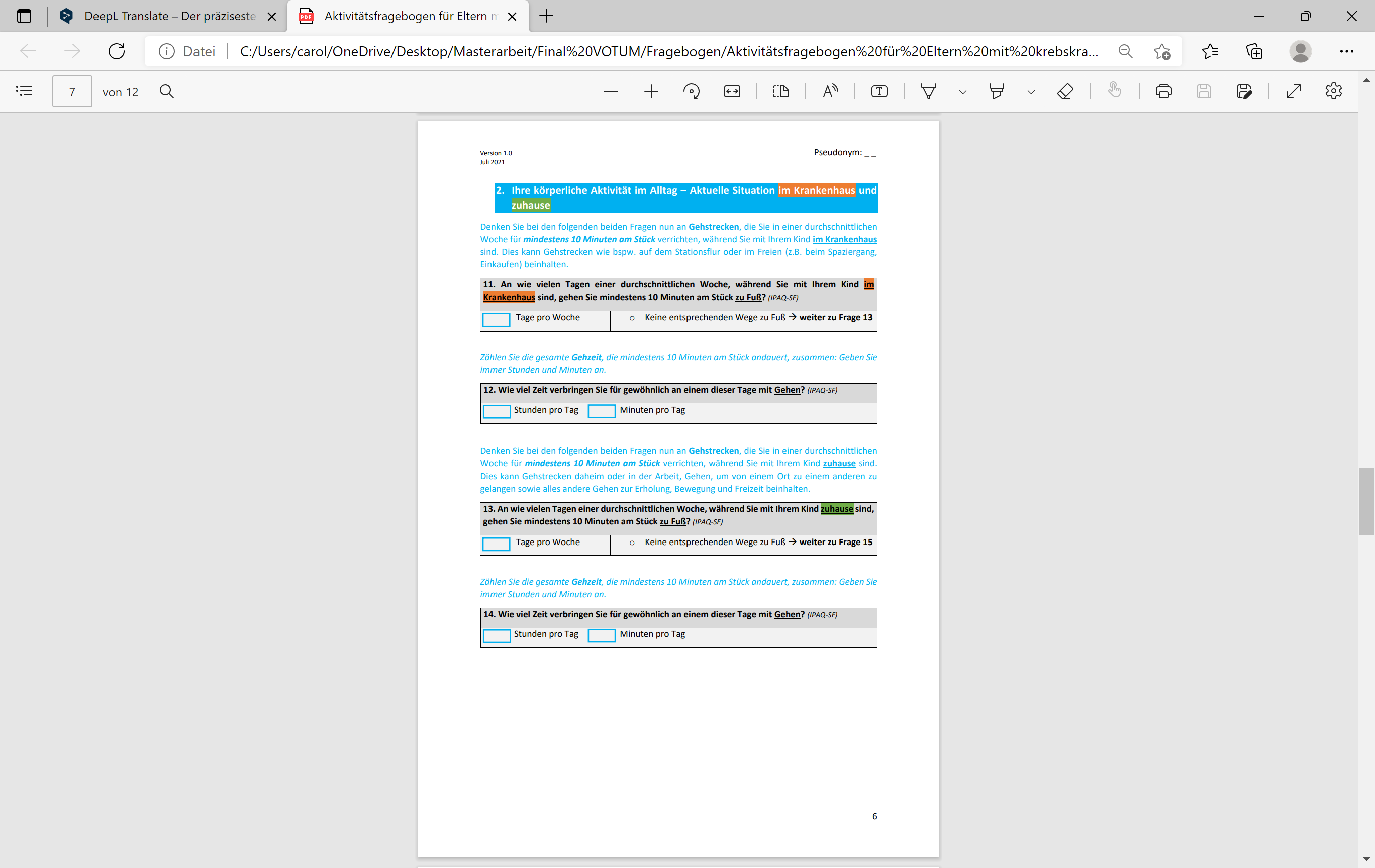


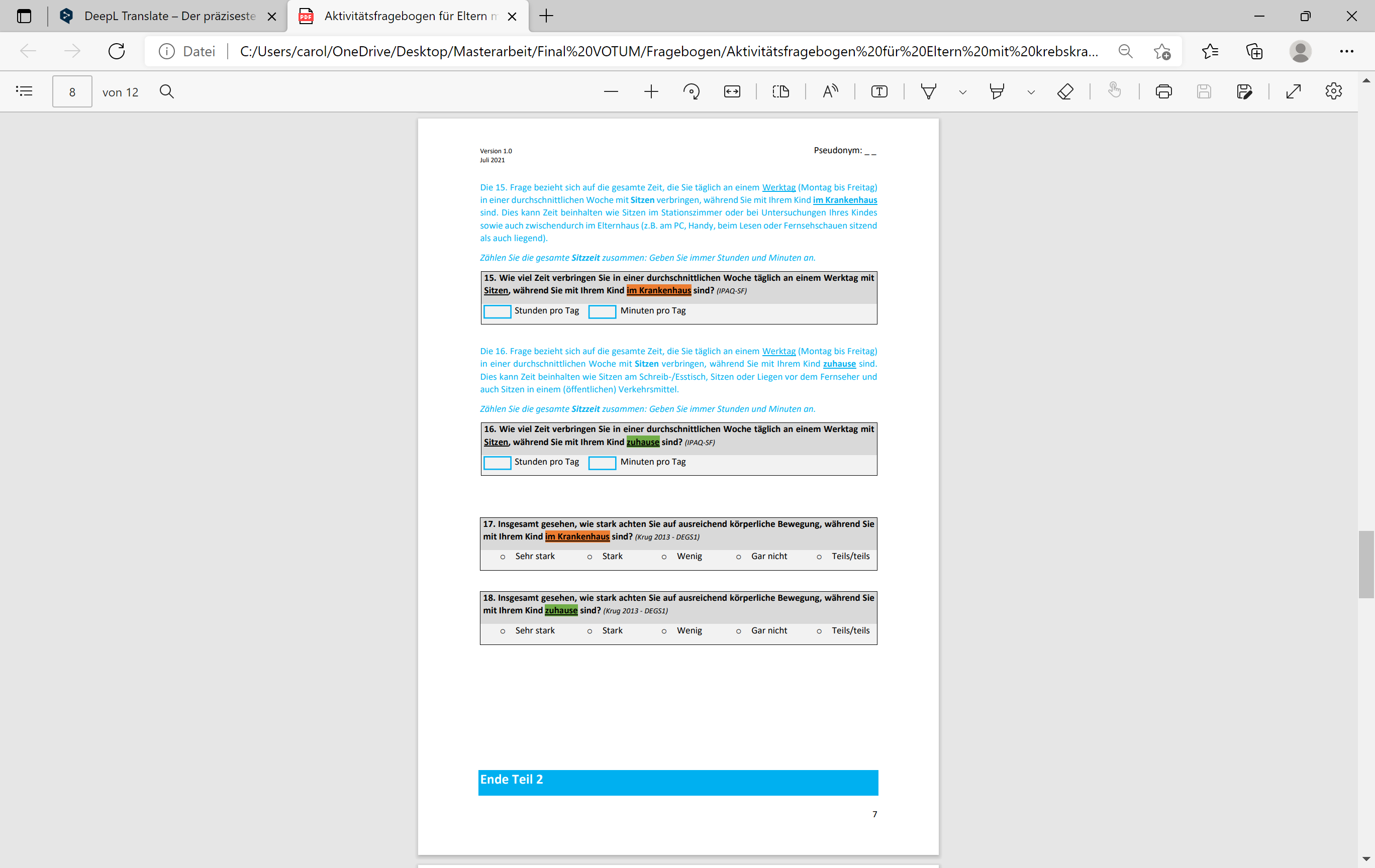


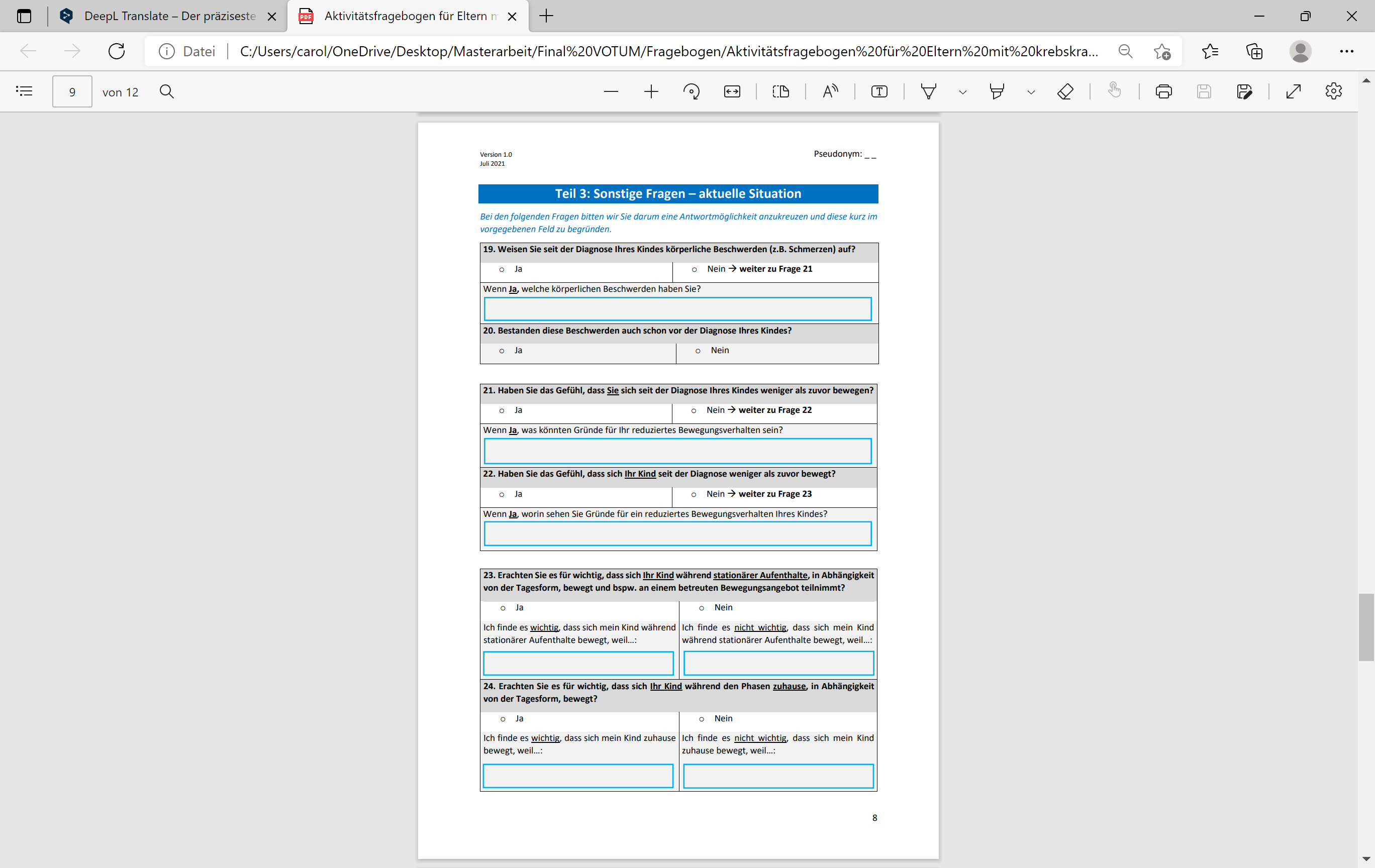


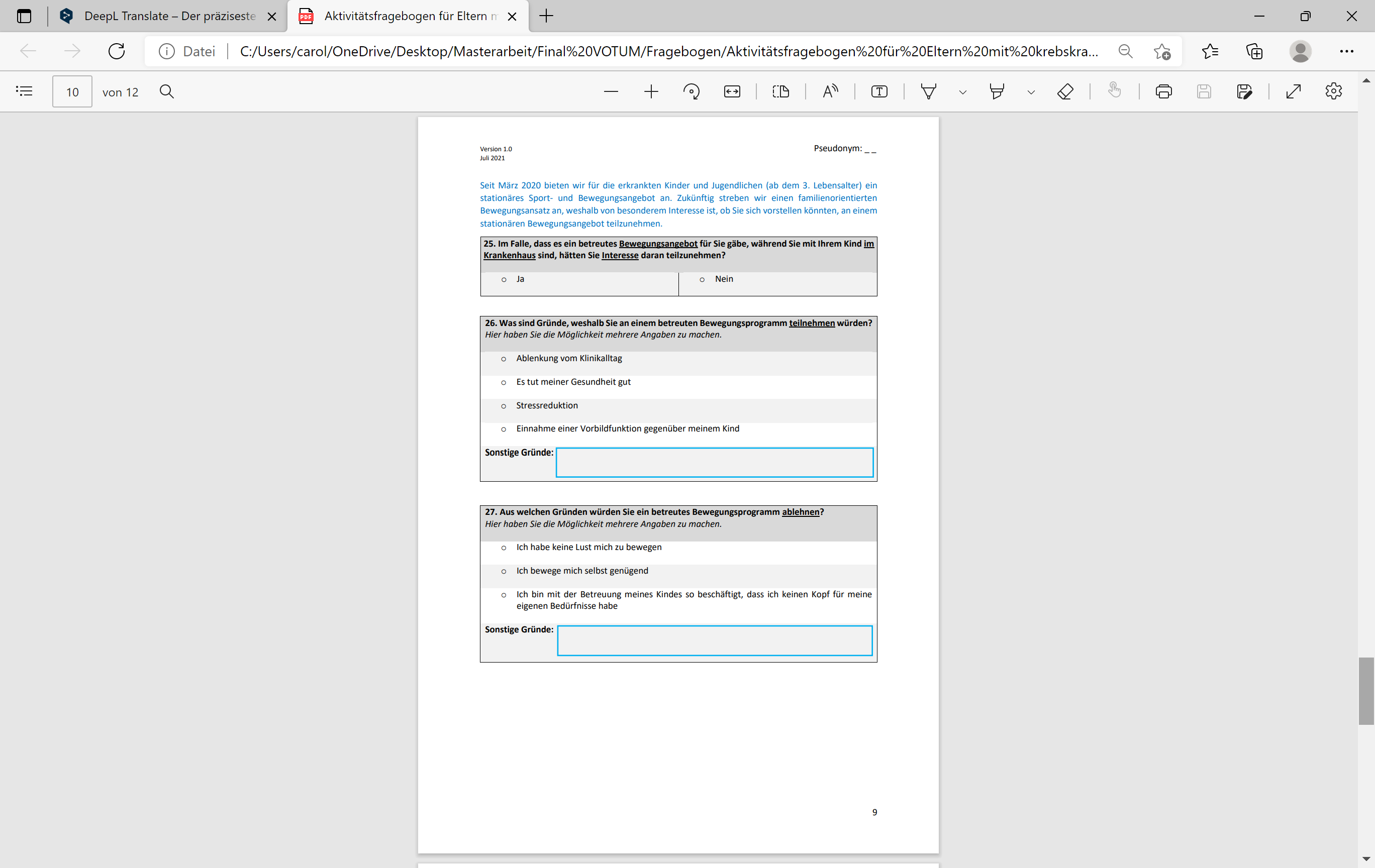


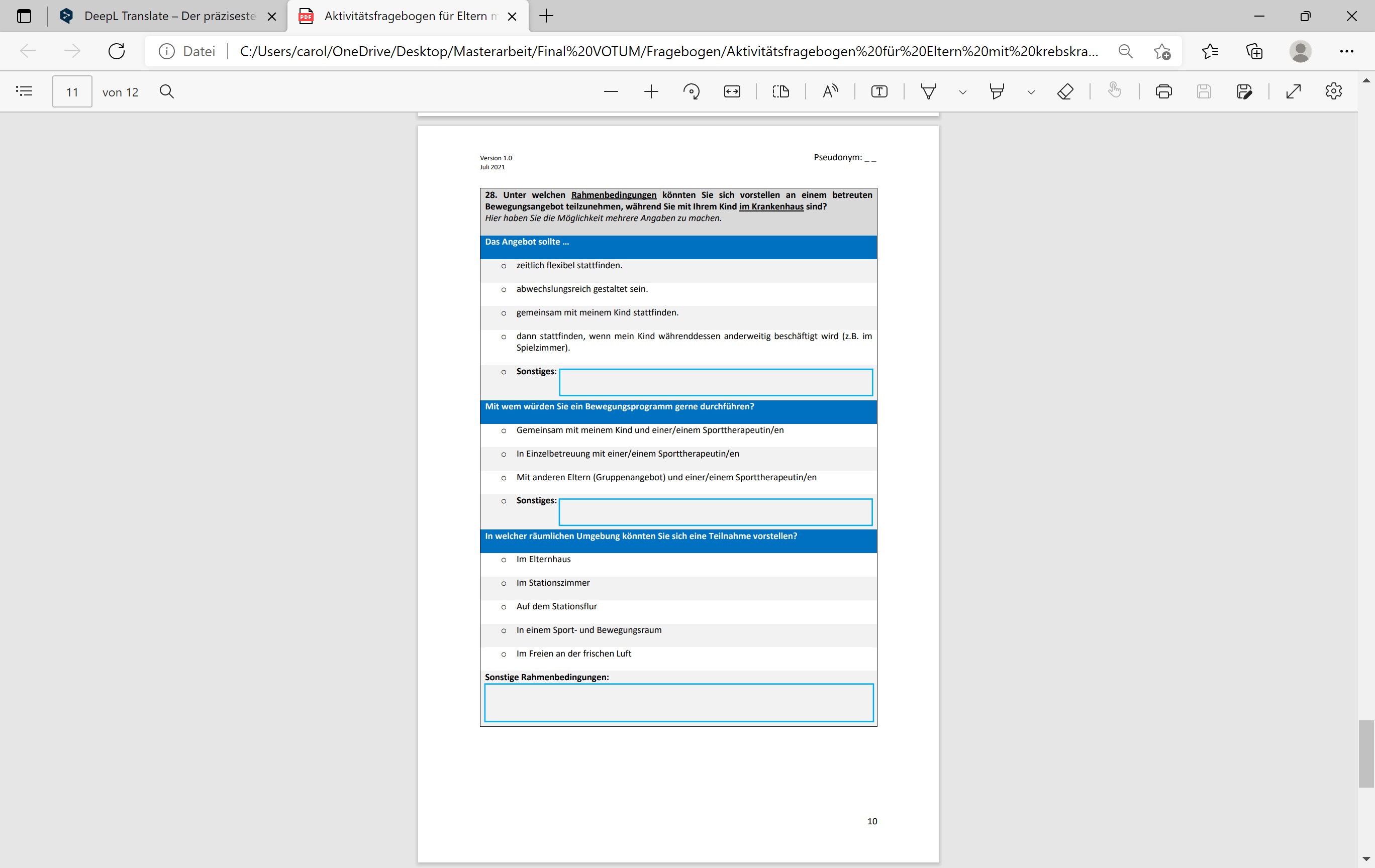


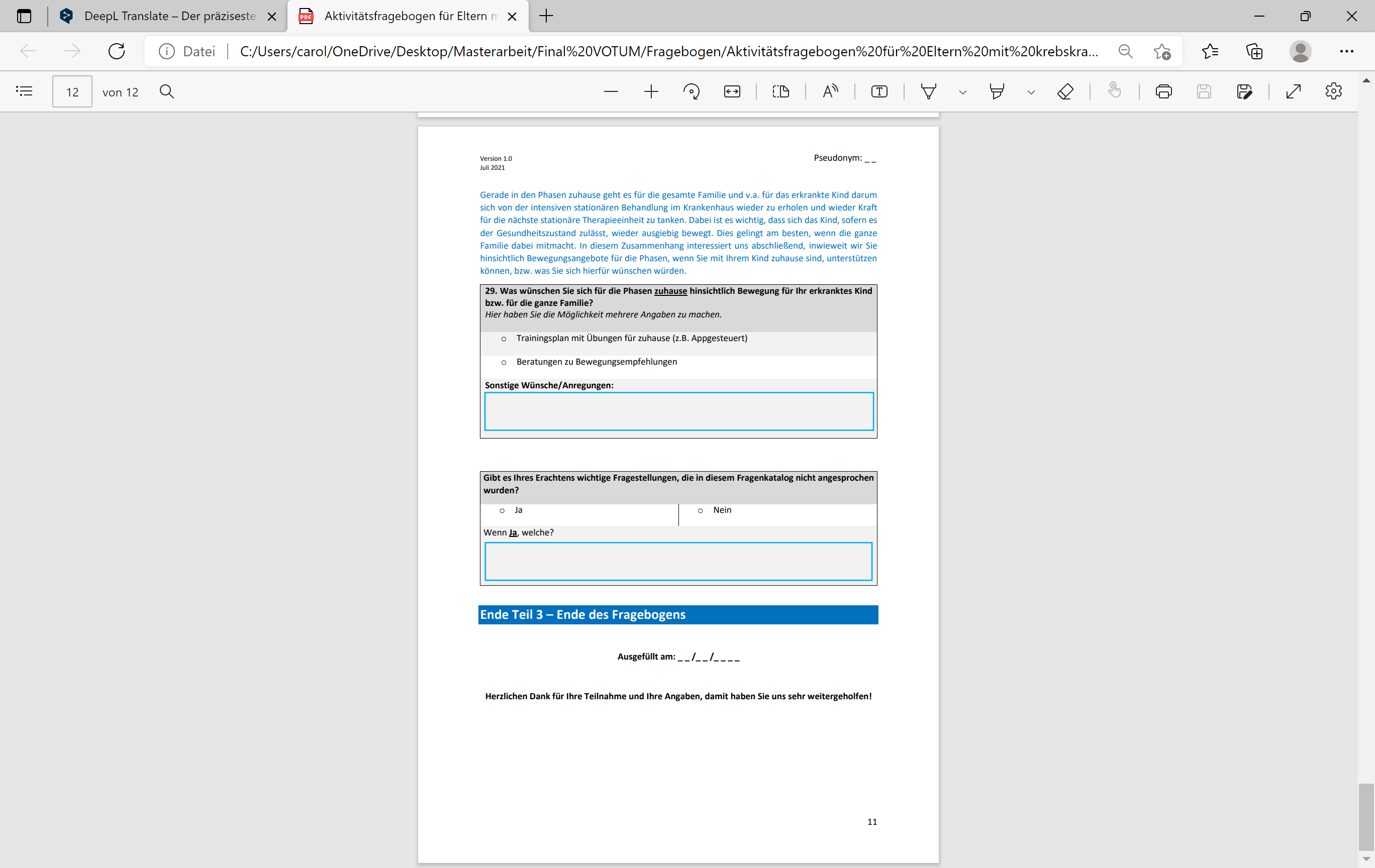


**Activity-Questionnaire:**

English version (not submitted and not reviewed by the Ethics Committee 🡪 no original document, just for review):


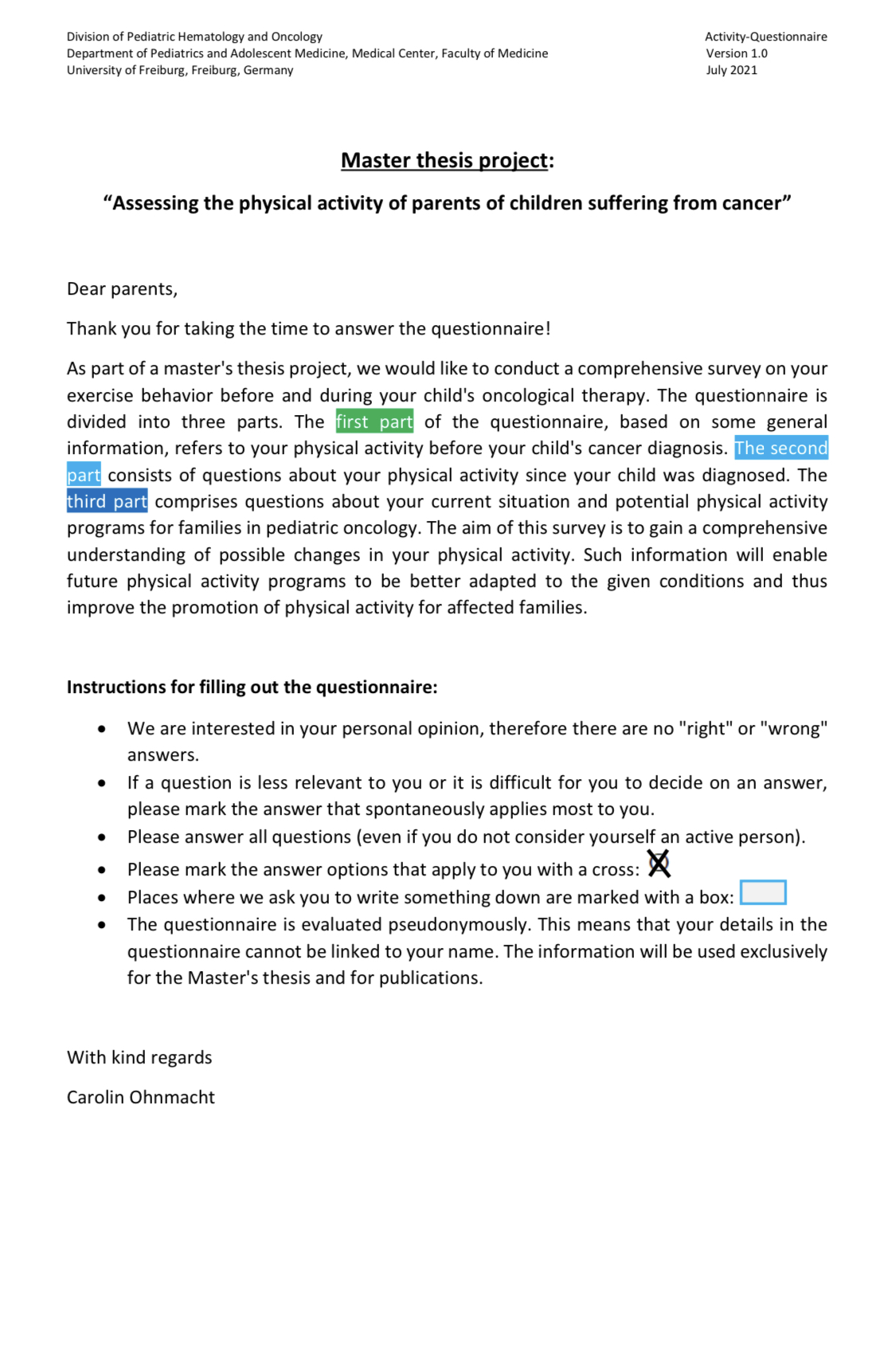


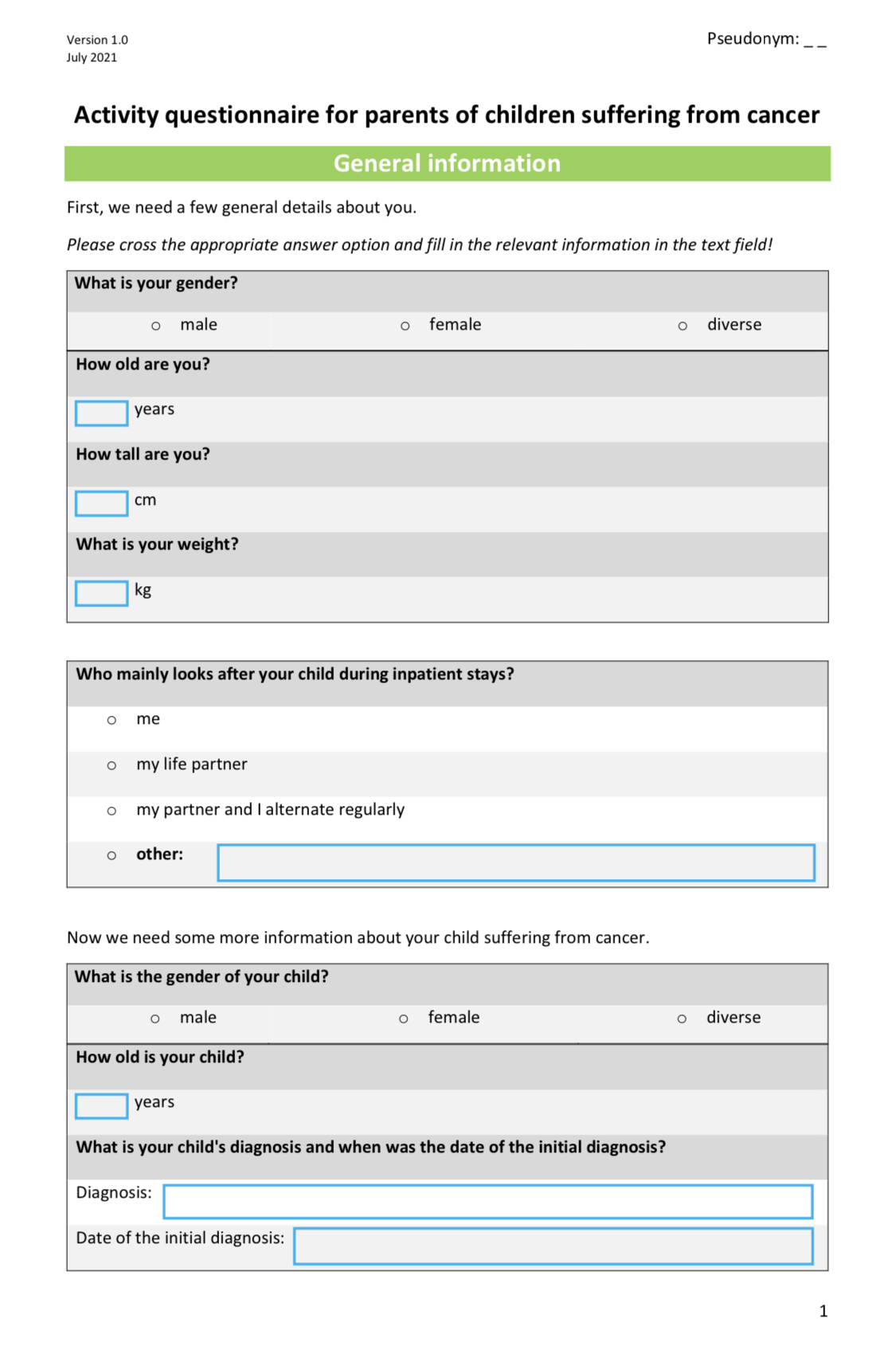


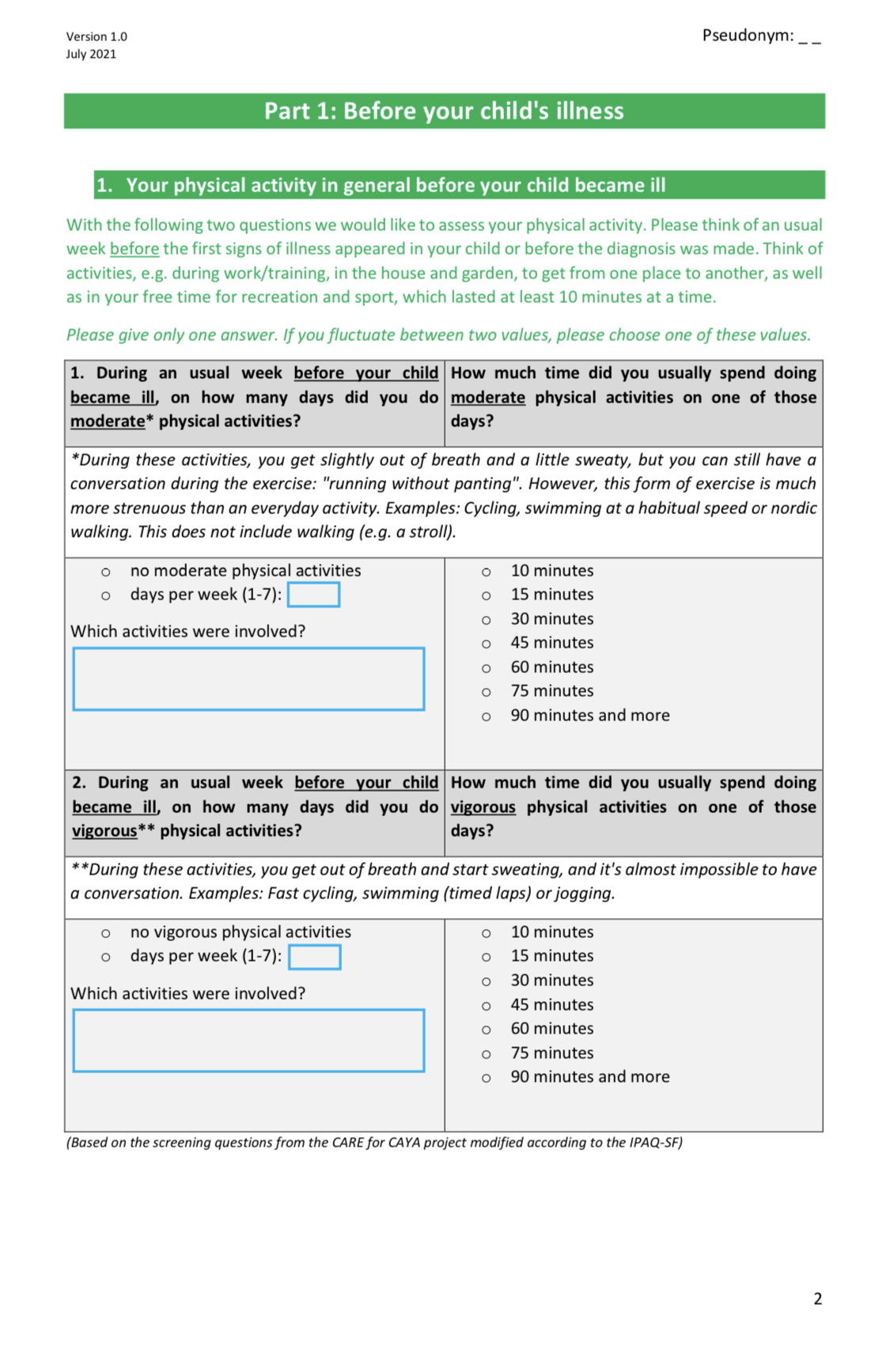


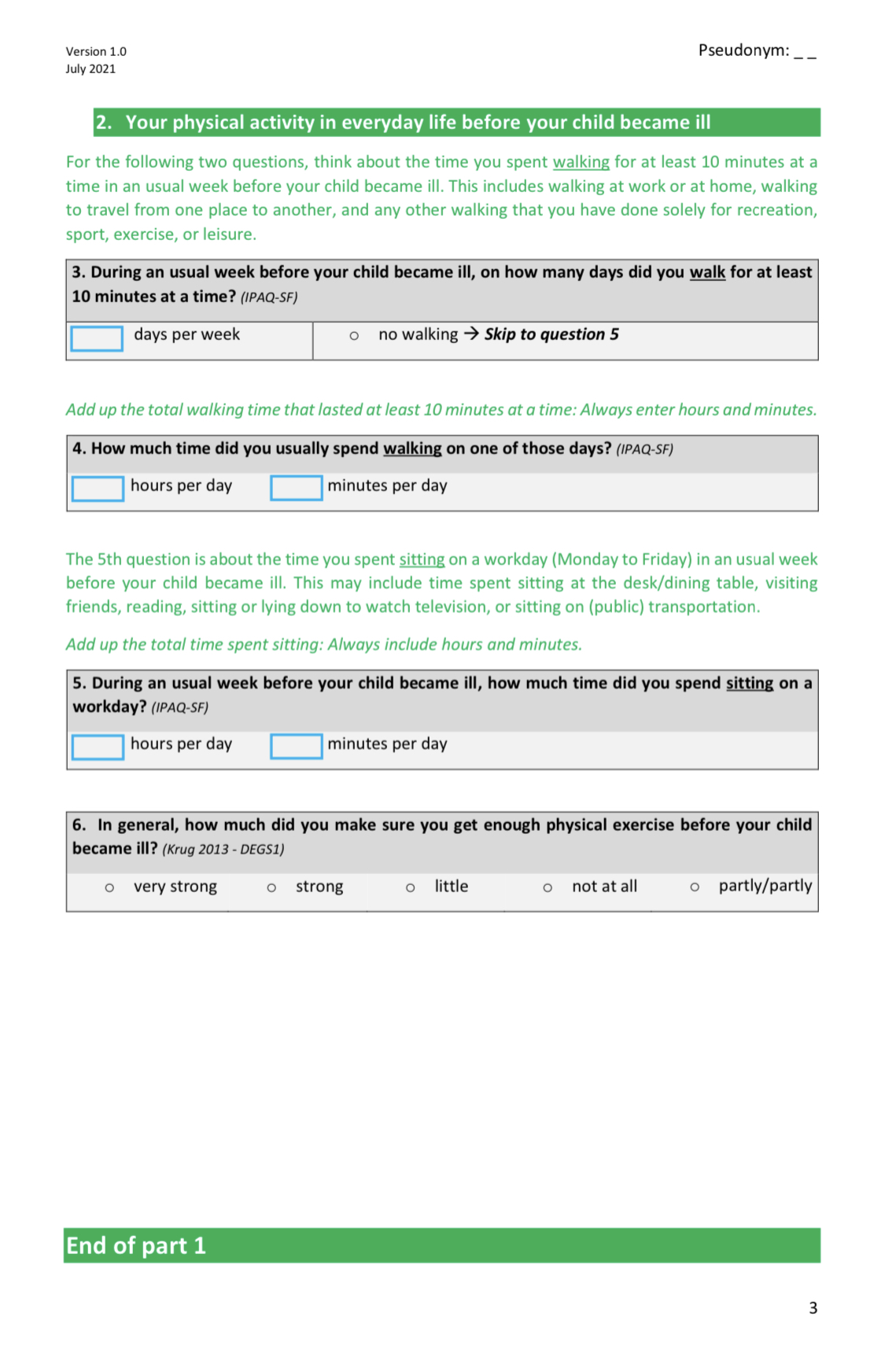


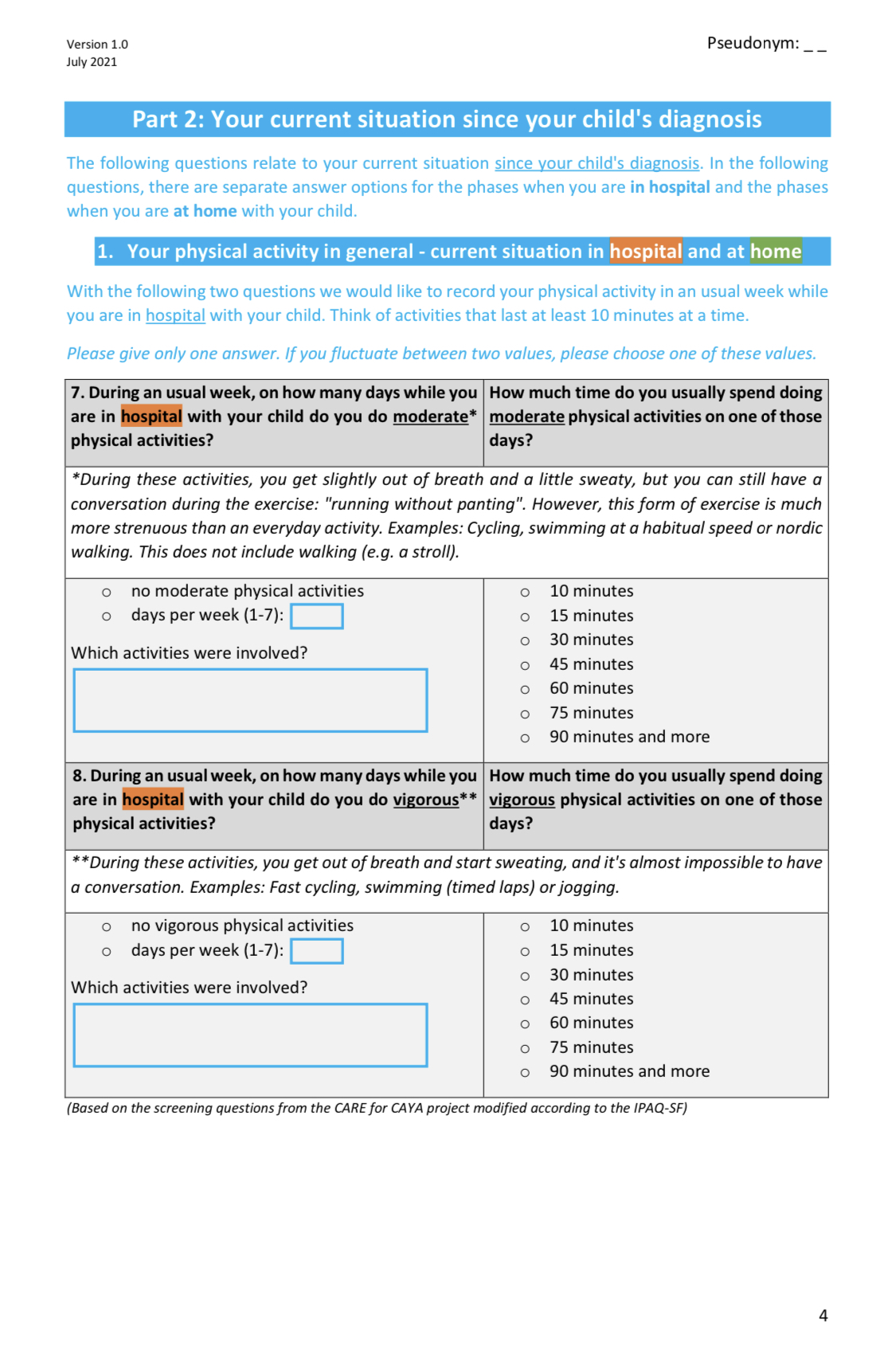


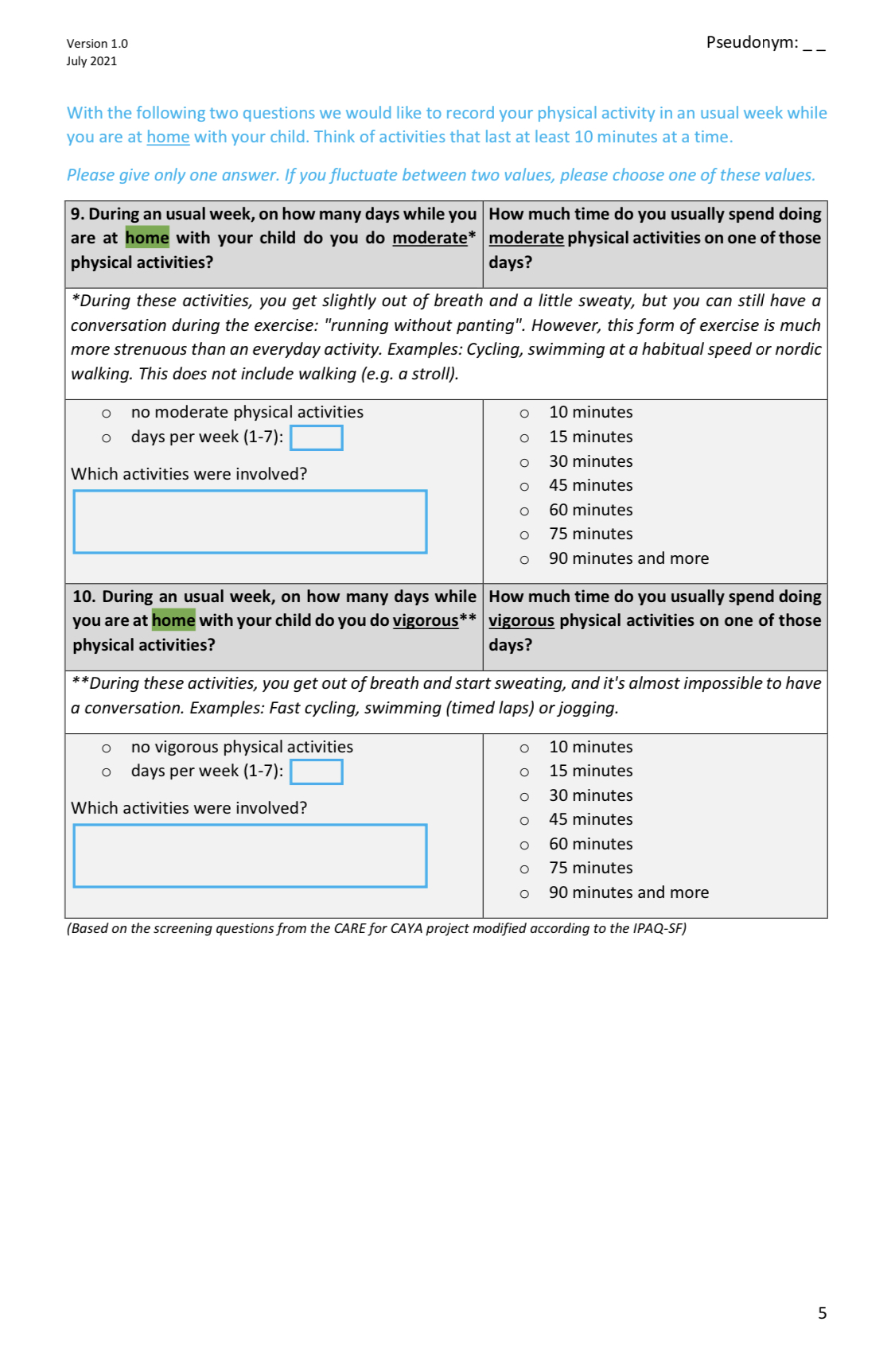


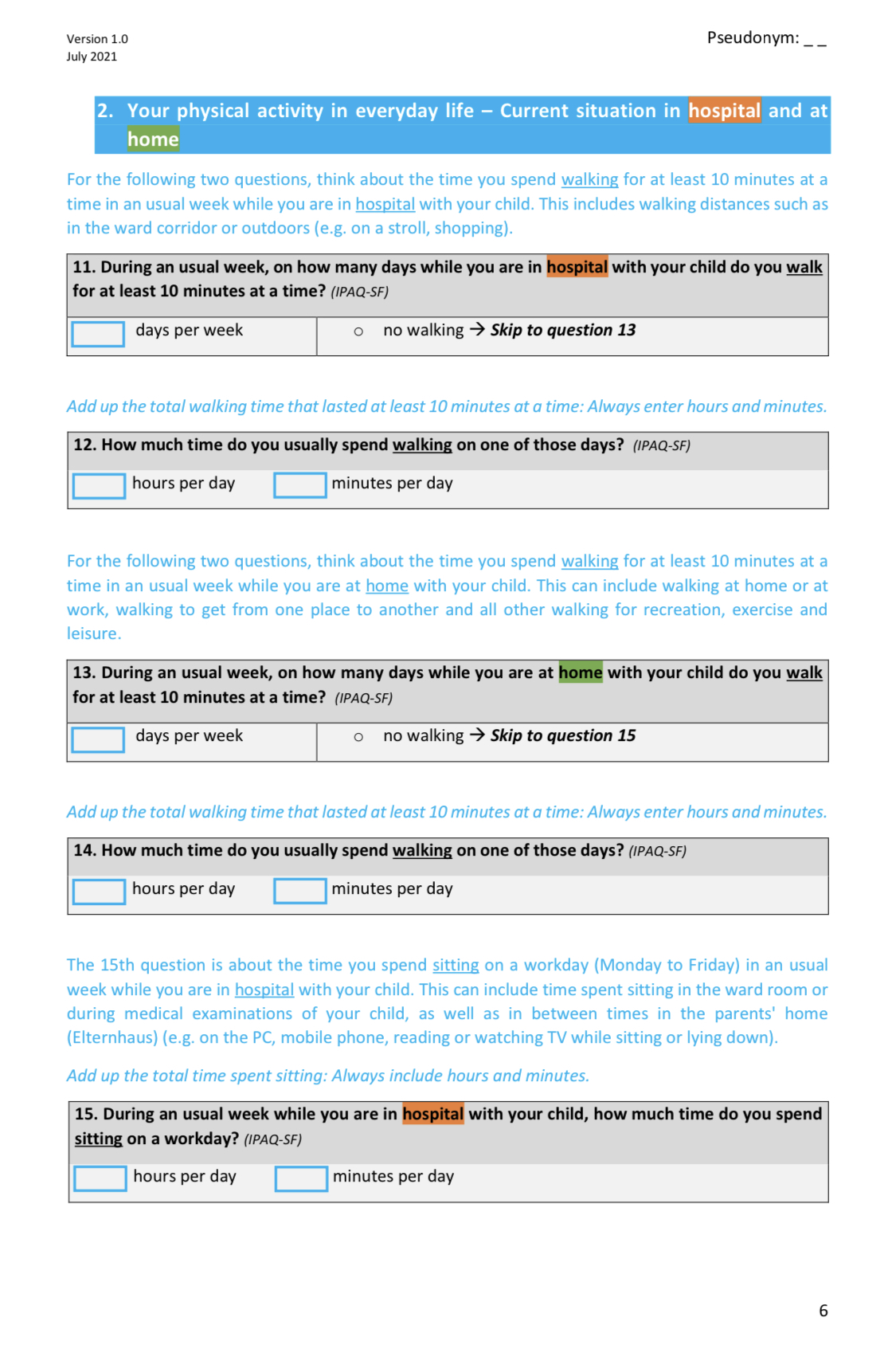


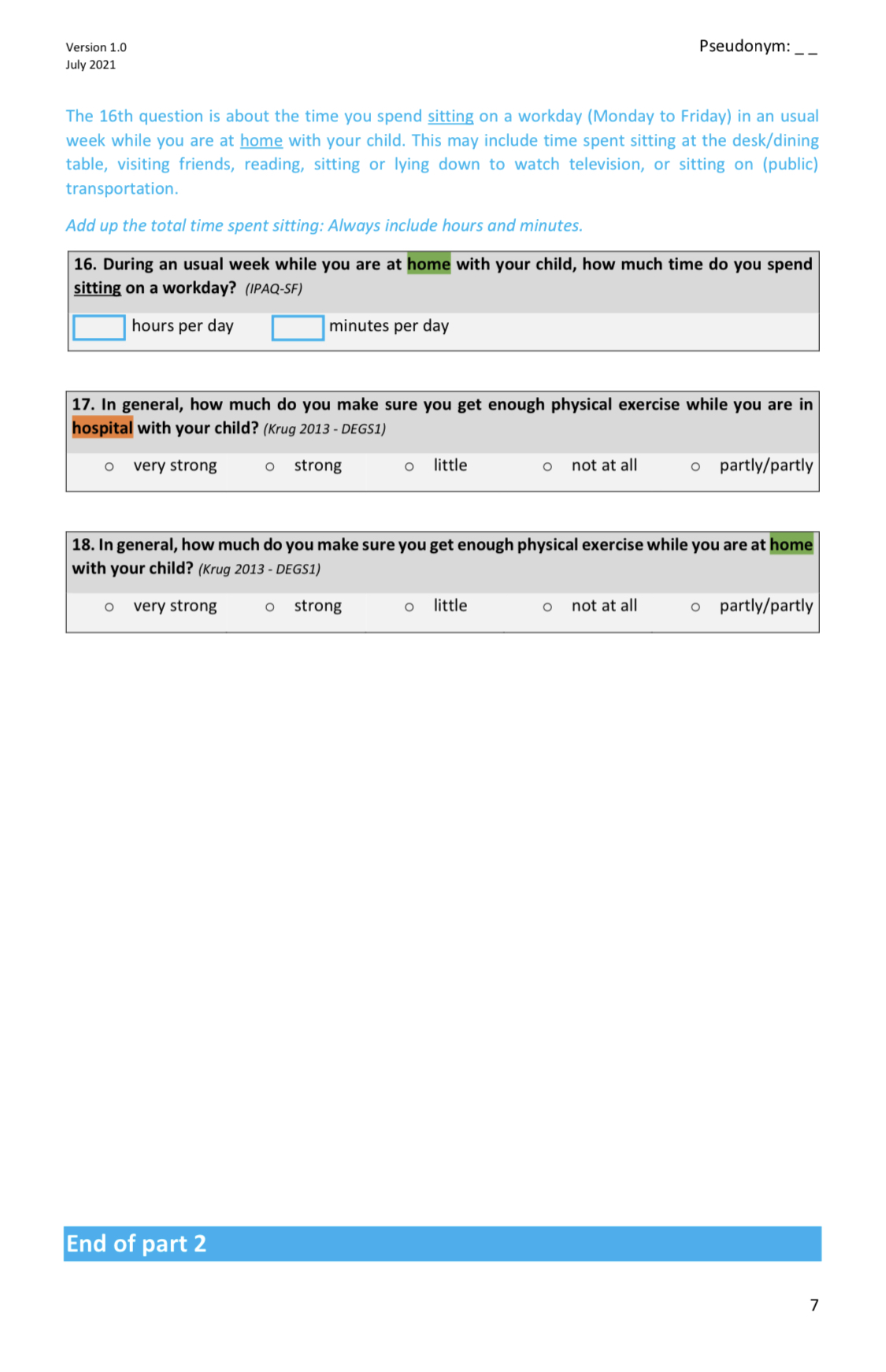


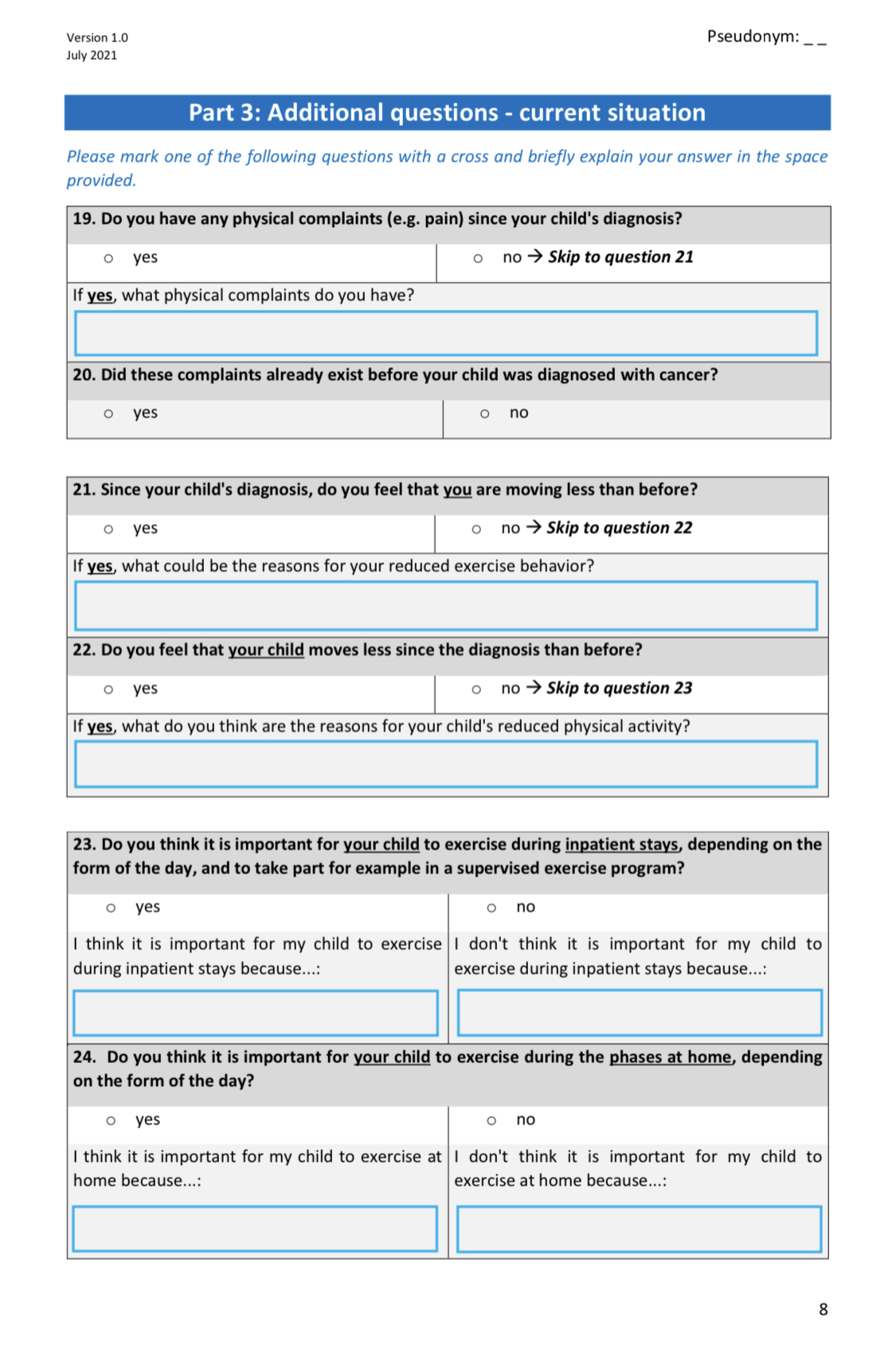


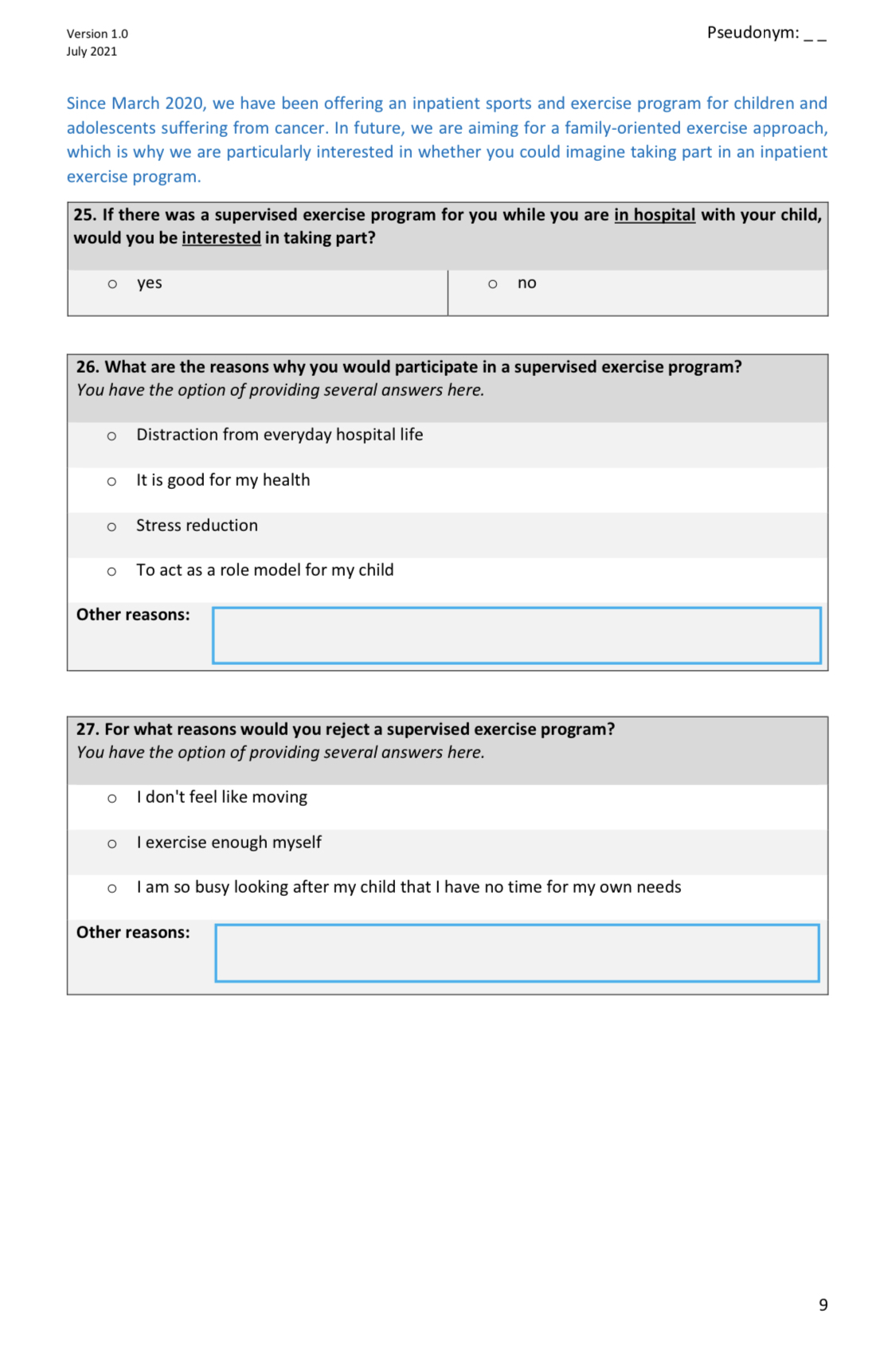


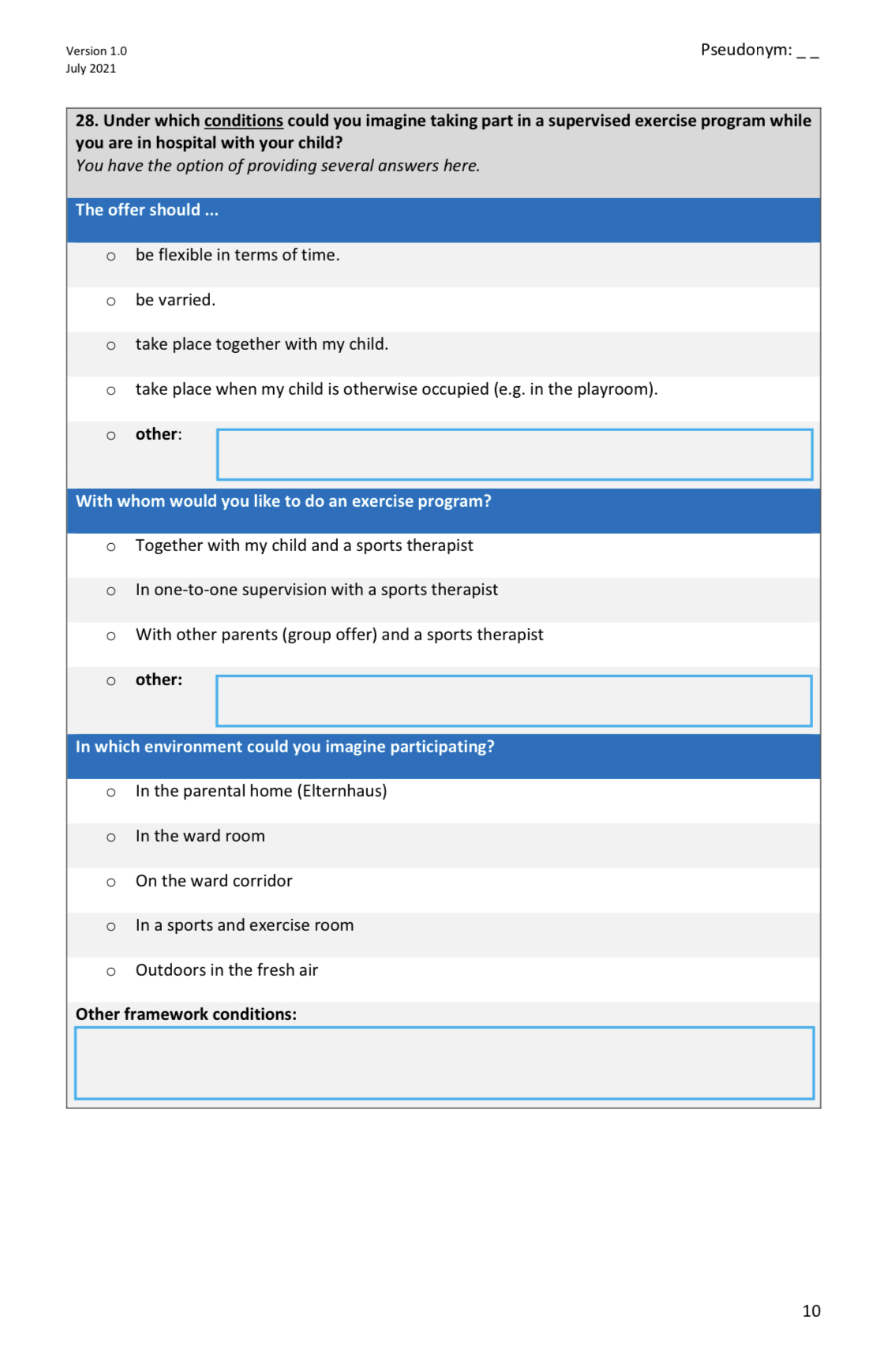


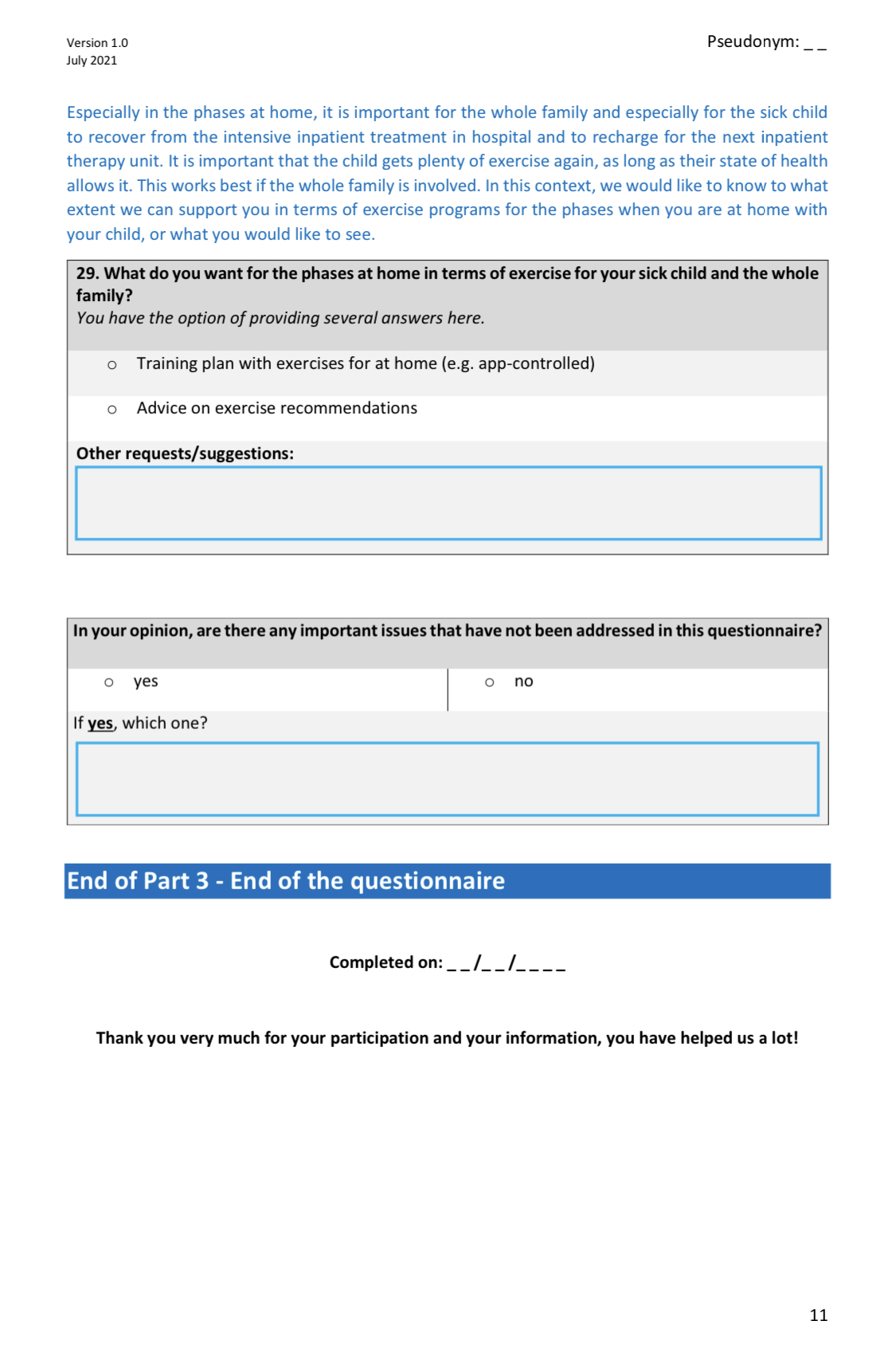

Supplement: Supplementary file 2 — Supplementary Material 2. [file 12889_2025_25455_MOESM2_ESM.docx]
