# Supplementary material for: Assessing the physical activity of parents of children suffering from cancer: a cross-sectional study
Source: BMC Public Health. 2025 Nov 17;25:3969. doi: 10.1186/s12889-025-25455-5 (PMC12621409; doi:10.1186/s12889-025-25455-5)
Supplement: Supplementary file 3 — Supplementary Material 3. [file 12889_2025_25455_MOESM3_ESM.docx]

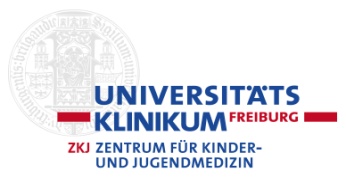


**Probandeninformation**

**Evaluation des Bewegungsverhaltes von Eltern mit krebskranken Kindern vor und während der onkologischen Intensivtherapie**

Sehr geehrte Eltern,

wir möchten Sie mit diesem Informationsblatt auf die Möglichkeit der Teilnahme an dem o.g. Forschungsprojekt hinweisen. Es handelt sich hierbei um ein wissenschaftliches Forschungsvorhaben der Klinik für Pädiatrische Hämatologie und Onkologie am Zentrum für Kinder- und Jugendmedizin des Universitätsklinikums Freiburg.

Diese schriftliche Information soll die wesentlichen Informationen vermitteln, damit Sie über die Teilnahme an diesem Forschungsvorhaben entscheiden können. Die Teilnahme ist freiwillig. Bevor Sie über Ihre Zustimmung zur Teilnahme entscheiden, ist es wichtig, dass Sie über Ihre Rechte und Pflichten informiert wurden. Bitte zögern Sie nicht, nach Bedenkzeit zu fragen, falls Sie dies möchten. Sofern Sie nicht an diesem Forschungsprojekt teilnehmen möchten, erwachsen Ihnen daraus keine Nachteile.

1. **Warum wird dieses Forschungsvorhaben durchgeführt?**

Regelmäßige körperliche Aktivität ist von elementarer Bedeutung für die Gesundheit. Dabei haben körperlich aktive Eltern sowie eine körperlich aktive Umgebung einen nachhaltigen Einfluss auf das Bewegungsverhalten von Kindern und Jugendlichen. Erkrankt das eigene Kind jedoch an Krebs, geraten herkömmliche Alltagsaktivitäten und Bewegungsmöglichkeiten bei allen Familienmitgliedern oftmals über mehrere Monate bis Jahre aus dem Gleichgewicht. Langwierige, intensive und überlebensnotwendige Behandlungen des Kindes, die mit mehrfachen stationären Aufenthalten verbunden sind, bestimmen plötzlich den Alltag der Familien.

Gerade während stationärer Behandlungsphasen erfahren die jungen Patienten eine deutliche Reduktion ihres Aktivitätsniveaus. Davon auszugehen ist, dass nicht nur die Patienten selbst, sondern ebenso Sie als Eltern, die ihre Kinder über die gesamte Zeit intensiv und kontinuierlich begleiten, von den therapie- und strukturbedingten Bewegungseinschränkungen betroffen sind. Während es in den letzten Jahren verstärkt zu einem Ausbau sporttherapeutischer Versorgungsstrukturen in pädiatrisch-onkologischen Behandlungszentren gekommen ist (u.a. seit März 2020 auch in Freiburg) erfahren Sie als Eltern, abgesehen von einer psychoonkologischen und sozialpädagogischen Betreuung, diesbezüglich bislang keine Beachtung. Besonders in dieser Zeit erscheint es aber wichtig, auch Ihre Gesundheit zu fördern und Sie nachhaltig zu befähigen, die körperliche Aktivität Ihrer Kinder zu unterstützen.

Vor diesem Hintergrund zielt dieses Forschungsvorhaben als erstes seiner Art darauf ab, das Bewegungsverhalten der Eltern vor und während der onkologischen Intensivtherapie des Kindes zu erfassen, um in einem späteren Schritt erstmals einen familienorientierten Ansatz in der Bewegungstherapie von Krebs betroffenen Familien zu begründen.

1. **Wie ist der Ablauf dieses Vorhabens?**

Eltern in einem Alter von 18-65 Jahren, die für eine Teilnahme infrage kommen, werden ab dem 2. stationären Aufenthalt oder nach mindestens 4-wöchiger stationärer Behandlung der Kinder in der pädiatrischen Hämatologie und Onkologie in Freiburg im Rahmen des bereits vorhandenen Bewegungsangebots für Kinder und Jugendliche rekrutiert. Die Studieninformation und Aufklärung erfolgt persönlich durch die Studienleitung, die auch das vorhandene Bewegungsangebot koordiniert. Bei Teilnahmeinteresse und Eignung erhalten Sie die zu unterzeichnende Einwilligungserklärung und den Fragebogen in Papierformat. Sie füllen dann einmalig einen Fragebogen aus. Das Ausfüllen des Fragebogens erfolgt freiwillig und pseudonymisiert. Die ausgefüllten Fragebögen werden in einem klinikinternen Ordner sowie zur Auswertung auf einem klinikinternen Server abgelegt. Die Analyse erhobener Daten erfolgt softwaregestützt (SPSS).

1. **Welchen persönlichen Nutzen oder welche Risiken haben Sie aufgrund der Teilnahme am Forschungsvorhaben?**

Durch die Teilnahme an dem Forschungsprojekt ist kein offensichtliches Risiko bekannt. Die Fragebogenstudie soll Basis für eine familienorientierte Bewegungstherapie in der Kinderonkologie in Freiburg sein, die dann zukünftigen Patienten zugutekommen kann. Die Fragebogenergebnisse werden Ihnen aber natürlich gerne zur Verfügung gestellt und erläutert, um Ihr individuelles Bewegungsverhalten zu verbessern.

1. **Kann die Zustimmung zur Teilnahme am Projekt jederzeit widerrufen werden?**

Die Teilnahme am Forschungsvorhaben ist freiwillig und setzt Ihre schriftliche Zustimmung voraus. Sie können jederzeit, auch ohne Angabe von Gründen, Ihre Teilnahme beenden, ohne dass Ihnen dadurch irgendwelche Nachteile entstehen. Sollten Ihre Daten bereits erhoben worden sein, werden nur Ihre personenbezogenen Daten aus der Auswertung gelöscht. Weitere Informationen zum Datenschutz finden Sie weiter unten. Sollten Sie Ihre Teilnahme widerrufen wollen, wenden Sie sich bitte an die Projektleitung.

1. **Was geschieht mit den erhobenen Daten?**

Mit Ihrem Einverständnis und Ihrer Unterschrift erlauben Sie uns, die im Rahmen der Studie gewonnenen Daten für wissenschaftliche Zwecke im Klinikum wie z.B. zur Weiterentwicklung der Bewegungstherapie in der Kinderonkologie und zur Forschung zu nutzen. Die Auswertung erhobener Daten erfolgt ausschließlich durch Studienpersonal. Ihre Daten unterliegen dabei der ärztlichen Schweigepflicht und werden in pseudonymisierter Form erhoben, d.h. mit einem Code versehen, der keinen Rückschluss auf Ihre Person erlaubt (ohne Angabe Ihres Namens oder Ihres Geburtsdatums). Somit werden Ihre Daten mit einer Vertraulichkeit behandelt, die über die kliniküblichen Datenschutzbestimmungen hinausgeht. Die Daten sind gegen unbefugten Zugriff gesichert und es erfolgt keine Weitergabe an Dritte. Im Falle einer Veröffentlichung der Studienergebnisse bleibt die Vertraulichkeit Ihrer persönlichen Daten ebenfalls unter Beachtung der datenschutzrechtlichen Bestimmungen gewährleistet.

1. **Welche weiteren Datenschutzrechte haben Sie?**

Im Rahmen der EU-Datenschutzverordnung (EU-DSGVO) sind wir nach Art. 13 und 14 der EU-DSGVO verpflichtet, Ihnen zum Zeitpunkt der Datenerhebung eine grundsätzliche Information zu Ihren Schutzrechten zu geben.

Als Rechtsgrundlage für die Datenverarbeitung nach Art. 6 Abs. 1a und Art. 9 Abs. 2a gilt Ihre Einwilligung. Im Rahmen der gesetzlichen Vorgaben können Sie Auskunft über Ihre gespeicherten Daten verlangen. Ebenso können Sie eine Berichtigung falscher Daten, eine Übertragung der zur Verfügung gestellten Daten sowie eine Löschung der Daten oder Einschränkung ihrer Verarbeitung verlangen.

Ihnen stehen sog. Betroffenenrechte zu, d.h. Rechte, die Sie als im Einzelfall betroffene Person ausüben können. Diese Rechte können Sie gegenüber dem örtlichen Studienleiter geltend machen in dessen Eigenschaft als im Rahmen der Studie für die Datenverarbeitung Verantwortlichem. (Kontaktdaten s. unten). Sie haben ein Recht auf Auskunft über Ihre im Rahmen des Forschungsvorhabens gespeicherten personenbezogenen Daten; hierzu gehört auch der Anspruch auf unentgeltliche Überlassung einer Kopie dieser Daten. Wenn Sie feststellen, dass unrichtige oder unvollständige Daten von Ihnen verarbeitet werden, können Sie eine Berichtigung bzw. Vervollständigung verlangen. Außerdem haben Sie unter bestimmten Voraussetzungen das Recht, die Löschung der Daten bzw. deren Anonymisierung zu verlangen.

Bei Anliegen zur Datenverarbeitung und zur Einhaltung des Datenschutzes können Sie sich auch an den Datenschutzbeauftragten wenden:

Universitätsklinikum Freiburg Datenschutzbeauftragter Breisacher Straße 153, 79110 Freiburg E-Mail: datenschutz@uniklinik-freiburg.de

Sie haben außerdem ein Beschwerderecht bei jeder Datenschutzaufsichtsbehörde. Eine Liste der Aufsichtsbehörden in Deutschland finden Sie unter:

https://www.bfdi.bund.de/DE/Infothek/Anschriften_Links/anschriften_links-node.htmlIm

1. **An wen können Sie sich bei weiteren Fragen wenden?**

Wenn Sie noch weitere Fragen haben, so wenden Sie sich bitte an den für das Forschungsvorhaben und die Datenverarbeitung verantwortlichen Projektleiter bzw. an die Stellvertreterin:

| **Leiter des Forschungsprojektes:**  UNIVERSITÄTSKLINIKUM FREIBURG  Zentrum für Kinder- und Jugendmedizin  Pädiatrische Hämatologie und Onkologie  **Dr. med. Alexander Puzik**  Funktionsoberarzt  Facharzt für Kinder- und Jugendheilkunde, SP Kinderhämatologie/-onkologie  Telefon: +49 761 270-45440  Telefax: +49 761 270-46160  E-Mail: alexander.puzik@uniklinik-freiburg.de | **Stellvertreterin des Forschungsprojektes:**  UNIVERSITÄTSKLINIKUM FREIBURG  Zentrum für Kinder- und Jugendmedizin  Pädiatrische Hämatologie und Onkologie  **Carolin Ohnmacht**  Sportwissenschaftlerin & Sporttherapeutin B.A.  E-Mail: carolin.ohnmacht@uniklinik-freiburg.de |
| --- | --- |


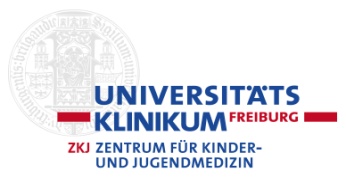


**Einwilligungserklärung**

**Evaluation des Bewegungsverhaltes von Eltern mit krebskranken Kindern vor und während der onkologischen Intensivtherapie**

Teilnehmer/in: ___________________________ Geburtsdatum: _____________________

(Name, Vorname) (DD/MM/YYYY)

Ich wurde ausreichend mündlich und schriftlich über das geplante wissenschaftliche Forschungsvorhaben informiert. Alle von mir im Zusammenhang mit diesem Vorhaben gestellten Fragen wurden zufriedenstellend beantwortet. Durch meine eigenhändige Unterschrift bestätige ich hiermit mein Einverständnis zur freiwilligen Teilnahme an diesem Forschungsprojekt.

Ich bin informiert worden, dass ich jederzeit die Einwilligung ohne Angaben von Gründen widerrufen kann. Hieraus entstehen mir und meinem Kind keine nachteiligen Folgen für die weitere medizinische Versorgung. Im Falle eines Widerrufs gebe ich meine Einwilligung, dass die bis dahin erhobenen Daten nach Löschung der personenbezogenen Daten (anonymisiert) für das Forschungsvorhaben weiterverwendet werden dürfen.

| **Information und Einwilligung zum Datenschutz:**  **Ich erkläre, dass ich informiert wurde und einwillige, dass im Rahmen des Projektes personenbezogene Daten, insbesondere Gesundheitsdaten, im Universitätsklinikum Freiburg, verschlüsselt aufgezeichnet, gespeichert und ausgewertet werden.**  Rechtsgrundlage für die Verarbeitung personenbezogener Daten ist gemäß Art. 9 Abs. 2a EU-DSGVO Ihre Einwilligung entsprechend dem Zweck dieses Forschungsvorhabens (beschrieben in Punkt 1).  Ich willige ein, dass im Zusammenhang mit diesem Forschungsprojekt Personen, die vom Leiter/Stellvertreter dieses Forschungsprojektes hierzu autorisiert wurden, zu Kontrollzwecken Einsicht in meine Krankenakte nehmen können. Für diese Maßnahme entbinde ich den Prüfarzt von der ärztlichen Schweigepflicht.  Ich willige ein, dass die Gesundheitsdaten bei mitbehandelnden Ärzten erhoben oder eingesehen werden, soweit dies für die ordnungsgemäße Durchführung und Überwachung des Forschungsprojektes notwendig ist. Insoweit entbinde ich diese Ärzte von der Schweigepflicht. |
| --- |

Ich willige ein, dass pseudonymisierte Daten an Kooperationspartner weitergeleitet und für wissenschaftliche Zwecke genutzt werden. Pseudonymisierte Form bedeutet, dass kein direkter Bezug zwischen den krankheitsbezogenen Daten und meinem Namen hergestellt werden kann. Ich willige auch ein, dass meine Daten in dieser Form zur Veröffentlichung von Publikationen verwendet werden dürfen.

Über meine Rechte hinsichtlich Auskunft, Berichtigung, Übertragung der von mir zur Verfügung gestellten Daten sowie Löschung der Daten oder Einschränkung ihrer Verarbeitung im Rahmen der EU-DSGVO wurde ich aufgeklärt. Diese Rechte können auf der Grundlage noch ausstehender Gesetzgebung des Landes Baden-Württemberg möglicherweise in zeitlicher und/oder inhaltlicher Hinsicht eingeschränkt werden, falls diese die wissenschaftlich korrekte Durchführung des Forschungsvorhabens unmöglich macht oder ernsthaft beeinträchtigt würde.

Eine Kopie dieser Teilnehmerinformation und Einwilligungserklärung habe ich erhalten.

__________________________ ______________ ___________________________________

Teilnehmer/in Datum Unterschrift

Der aufklärende Arzt bestätigt mit seiner Unterschrift, dass er das Aufklärungsgespräch geführt und die Einwilligung des Probanden eingeholt hat.

__________________________ ______________ __________________________________

Name des aufklärenden Arztes Datum Unterschrift
