# Supplementary material for: Assessing the physical activity of parents of children suffering from cancer: a cross-sectional study
Source: BMC Public Health. 2025 Nov 17;25:3969. doi: 10.1186/s12889-025-25455-5 (PMC12621409; doi:10.1186/s12889-025-25455-5)
Supplement: Supplementary file 4 — Supplementary Material 4. [file 12889_2025_25455_MOESM4_ESM.docx]

**Masterarbeitsprojekt:**

**Evaluation des Bewegungsverhaltens von Eltern mit krebskranken Kindern vor und während der onkologischen Intensivtherapie**

Sehr geehrte Eltern,

Vielen Dank, dass Sie sich für die Beantwortung des Fragebogens Zeit nehmen!

Im Rahmen eines Masterarbeitsprojekts möchten wir eine umfassende Befragung zu Ihrem Bewegungsverhalten vor und während der onkologischen Intensivtherapie Ihres Kindes durchführen. Der Fragebogen gliedert sich in drei Teile. Der erste Teil des Fragebogens bezieht sich nach einigen allgemeinen Angaben auf Ihre körperliche Aktivität **vor der Erkrankung Ihres Kindes bzw. bevor die Diagnose vorlag**. Im zweiten Teil des Fragebogens werden Fragen zu Ihrer körperlichen Aktivität **seit der Diagnose Ihres Kindes** gestellt. Der dritte Teil besteht aus Fragen zu Ihrer **aktuellen Situation und zur Ausgestaltung eines möglichen Bewegungsangebots für Familien in der Kinderonkologie**. Durch diese Befragung soll ein umfassender Einblick in mögliche Veränderungen Ihrer körperlichen Aktivität gewonnen werden. Zukünftige Bewegungsprogramme können dann besser auf die gegebenen Bedingungen angepasst und damit die Bewegungsförderung betroffener Familien verbessert werden.

**Hinweise zum Ausfüllen des Fragebogens:**

- Wir sind an Ihrer persönlichen Meinung interessiert, weshalb es keine „richtigen“ oder „falschen“ Antworten gibt.
- Falls eine Frage weniger auf Sie zutreffen sollte oder es Ihnen schwerfällt, sich für eine Antwort zu entscheiden, kreuzen Sie bitte die Antwort an, die spontan am ehesten auf Sie zutrifft.
- Bitte beantworten Sie alle Fragen (auch wenn Sie sich nicht als aktive Person ansehen).
- Bitte markieren Sie die auf Sie zutreffenden Antwortmöglichkeiten mit einem Kreuz:
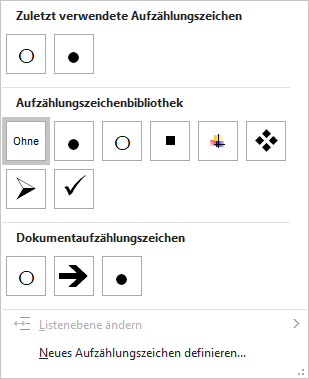

- Stellen, an denen wir Sie bitten, etwas aufzuschreiben, sind durch einen Kasten:

gekennzeichnet.

- Der Fragebogen wird pseudonymisiert ausgewertet. Ihre Angaben im Fragebogen können also nicht mit Ihrem Namen in Verbindung gebracht werden. Die Angaben werden ausschließlich im Rahmen der Masterarbeit und für Veröffentlichungen/Publikationen verwendet.

Mit freundlichen Grüßen

Carolin Ohnmacht (Sportwissenschaftlerin und Sporttherapeutin B.A.)

**Aktivitätsfragebogen für Eltern mit krebskranken Kindern**

**Allgemeine Angaben**

Vorab benötigen wir ein paar allgemeine Angaben zu Ihnen.

*Bitte kreuzen Sie die passende Antwortmöglichkeit an und ergänzen Sie die jeweiligen Angaben im Textfeld!*

| **Was ist Ihr Geschlecht?** | | |
| --- | --- | --- |
| - Männlich | - Weiblich | - Divers |

| **Wie alt sind Sie?** |
| --- |
| Jahre |
| **Wie groß sind Sie?** |
| cm |
| **Was ist Ihr Gewicht?** |
| kg |

| **Wer betreut Ihr Kind hauptsächlich während stationärer Behandlungen?** |
| --- |
| - Ich |
| - Mein/e Lebenspartner/in |
| - Mein/e Lebenspartner/in und ich wechseln uns regelmäßig ab |
| - **Sonstiges:** |

Nun benötigen wir noch ein paar Informationen zu Ihrem erkrankten Kind.

| **Was ist das Geschlecht Ihres Kindes?** | | |
| --- | --- | --- |
| - Männlich | - Weiblich | - Divers |

| **Wie alt ist Ihr Kind?** |
| --- |
| Jahre |
| **Welche Diagnose liegt bei Ihrem Kind vor und wann war das Datum der Erstdiagnose?** |
| Diagnose: |
| Datum der Erstdiagnose: |

**Teil 1: Vor der Erkrankung Ihres Kindes**

1. **Ihre körperliche Aktivität im Allgemeinen, bevor Ihr Kind erkrankt ist**

Mit den folgenden beiden Fragen möchten wir gerne Ihre körperliche Aktivität erfassen. Bitte denken Sie bei der Beantwortung der Fragen an eine durchschnittliche Woche, **bevor erste Krankheitszeichen bei Ihrem Kind auftraten bzw. bevor die Diagnose vorlag**. Denken Sie sowohl an die Aktivitäten z.B. während der Arbeit/Ausbildung, in Haus und Garten, um von einem Ort zum anderen zu kommen als auch in Ihrer Freizeit für Erholung und Sport, die ***mindestens 10 Minuten am Stück*** andauerten.

*Bitte machen Sie nur eine Angabe. Schwanken Sie zwischen zwei Werten, entscheiden Sie sich bitte für einen dieser Werte.*

| **1. An wie vielen Tagen einer durchschnittlichen Woche, bevor Ihr Kind erkrankt ist, haben Sie etwas anstrengende* Aktivitäten ausgeübt?** | **Wie lange sind Sie an diesen Tagen durchschnittlich körperlich aktiv gewesen?** |
| --- | --- |
| **Bei diesen Aktivitäten kommt man leicht außer Atem und etwas ins Schwitzen, aber man kann sich während der Belastung noch unterhalten: „Laufen, ohne zu schnaufen“. Allerdings ist diese Form der Bewegung deutlich anstrengender als eine Alltagsaktivität. Beispiele: Radfahren, Schwimmen bei gewöhnlicher Geschwindigkeit oder Walken. Hierzu zählt nicht das zu Fuß gehen (z.B. Spaziergang).* | |
| - An keinem Tag - Anzahl der Tage (1-7):   Um welche Aktivität/en handelte es sich dabei? | - 10 Minuten - 15 Minuten - 30 Minuten - 45 Minuten - 60 Minuten - 75 Minuten - 90 Minuten und mehr |
| **2. An wie vielen Tagen einer durchschnittlichen Woche, bevor Ihr Kind erkrankt ist, haben Sie anstrengende** Aktivitäten ausgeübt?** | **Wie lange sind Sie an diesen Tagen durchschnittlich körperlich aktiv gewesen?** |
| ***Bei diesen Aktivitäten kommt man außer Atem und ins Schwitzen, man kann sich währenddessen kaum noch unterhalten. Beispiele: Schnelles Radfahren, Schwimmen (Bahnen auf Zeit) oder Joggen.* | |
| - An keinem Tag - Anzahl der Tage (1-7):   Um welche Aktivität/en handelte es sich dabei? | - 10 Minuten - 15 Minuten - 30 Minuten - 45 Minuten - 60 Minuten - 75 Minuten - 90 Minuten und mehr |

*(In Anlehnung an die Screeningfragen aus dem CARE for CAYA Projekt modifiziert nach dem IPAQ-SF)*

1. **Ihre körperliche Aktivität im Alltag, bevor Ihr Kind erkrankt ist**

Denken Sie bei den folgenden beiden Fragen nun an **Gehstrecken**, die Sie in einer durchschnittlichen Woche, bevor Ihr Kind erkrankt ist, für ***mindestens 10 Minuten*** ***am Stück*** verrichtet haben. Dies kann Gehstrecken daheim oder in der Arbeit, Gehen, um von einem Ort zu einem anderen zu gelangen sowie alles andere Gehen zur Erholung, Bewegung und Freizeit beinhalten.

| **3. An wie vielen Tagen einer durchschnittlichen Woche, bevor Ihr Kind erkrankt ist, sind Sie mindestens 10 Minuten am Stück zu Fuß gegangen?** *(IPAQ-SF)* | |
| --- | --- |
| Tage pro Woche | - Keine entsprechenden Wege zu Fuß 🡪 **weiter zu Frage 5** |

*Zählen Sie die gesamte* ***Gehzeit****, die* ***mindestens 10 Minuten am Stück*** *andauerte, zusammen:* *Geben Sie immer Stunden und Minuten an.*

| **4. Wie viel Zeit haben Sie für gewöhnlich an einem dieser Tage mit Gehen verbracht?** *(IPAQ-SF)* |
| --- |
| Stunden pro Tag Minuten pro Tag |

Die 5. Frage bezieht sich auf die gesamte Zeit, die Sie täglich an einem Werktag (Montag bis Freitag) in einer durchschnittlichen Woche, bevor Ihr Kind erkrankt ist, mit **Sitzen** verbracht haben. Dies kann Zeit beinhalten wie Sitzen am Schreib-/Esstisch, Besuchen von Freunden, Sitzen oder Liegen vor dem Fernseher und auch Sitzen in einem (öffentlichen) Verkehrsmittel.

*Zählen Sie die gesamte* ***Sitzzeit*** *zusammen:* *Geben Sie immer Stunden und Minuten an.*

| **5. Wie viel Zeit haben Sie in einer durchschnittlichen Woche täglich an einem Werktag mit Sitzen verbracht, bevor Ihr Kind erkrankt ist?** *(IPAQ-SF)* |
| --- |
| Stunden pro Tag Minuten pro Tag |

| **6. Insgesamt gesehen, wie stark haben Sie auf ausreichend körperliche Bewegung geachtet, bevor Ihr Kind erkrankt ist?** *(Krug 2013 - DEGS1)* | | | | |
| --- | --- | --- | --- | --- |
| - Sehr stark | - Stark | - Wenig | - Gar nicht | - Teils/teils |

**Ende Teil 1**

**Teil 2: Ihre aktuelle Situation seit der Diagnose Ihres Kindes**

Die folgenden Fragen beziehen sich nun auf Ihre **aktuelle Situation, seit der Diagnose Ihres Kindes**. Bei den folgenden Fragen gibt es getrennte Antwortmöglichkeiten für die Phasen, während Sie mit Ihrem Kind **im Krankenhaus** und die Phasen, in denen Sie mit Ihrem Kind **zuhause** sind.

1. **Ihre körperliche Aktivität im Allgemeinen – Aktuelle Situation im Krankenhaus und zuhause**

Mit den folgenden beiden Fragen möchten wir gerne Ihre körperliche Aktivität in einer durchschnittlichen Woche erfassen, während Sie mit Ihrem Kind **im Krankenhaus** sind. Denken Sie an Aktivitäten, die ***mindestens* *10 Minuten am Stück*** andauern.

*Bitte machen Sie nur eine Angabe. Schwanken Sie zwischen zwei Werten, entscheiden Sie sich bitte für einen dieser Werte.*

| **7. An wie vielen Tagen einer durchschnittlichen Woche, während Sie mit Ihrem Kind im Krankenhaus sind, üben Sie etwas anstrengende* Aktivitäten aus?** | **Wie lange sind Sie an diesen Tagen durchschnittlich körperlich aktiv?** |
| --- | --- |
| **Bei diesen Aktivitäten kommt man leicht außer Atem und etwas ins Schwitzen, aber man kann sich während der Belastung noch unterhalten: „Laufen, ohne zu schnaufen“. Allerdings ist diese Form der Bewegung deutlich anstrengender als eine Alltagsaktivität. Beispiele: Radfahren, Schwimmen bei gewöhnlicher Geschwindigkeit oder Walken. Hierzu zählt nicht das zu Fuß gehen (z.B. Spaziergang).* | |
| - An keinem Tag - Anzahl der Tage (1-7):   Um welche Aktivität/en handelt es sich dabei? | - 10 Minuten - 15 Minuten - 30 Minuten - 45 Minuten - 60 Minuten - 75 Minuten - 90 Minuten und mehr |
| **8. An wie vielen Tagen einer durchschnittlichen Woche, während Sie mit Ihrem Kind im Krankenhaus sind, üben Sie anstrengende** Aktivitäten aus?** | **Wie lange sind Sie an diesen Tagen durchschnittlich körperlich aktiv?** |
| ***Bei diesen Aktivitäten kommt man außer Atem und ins Schwitzen, man kann sich währenddessen kaum noch unterhalten. Beispiele: Schnelles Radfahren, Schwimmen (Bahnen auf Zeit) oder Joggen.* | |
| - An keinem Tag - Anzahl der Tage (1-7):   Um welche Aktivität/en handelt es sich dabei? | - 10 Minuten - 15 Minuten - 30 Minuten - 45 Minuten - 60 Minuten - 75 Minuten - 90 Minuten und mehr |

*(In Anlehnung an die Screeningfragen aus dem CARE for CAYA Projekt modifiziert nach dem IPAQ-SF)*

Mit den folgenden beiden Fragen möchten wir gerne Ihre körperliche Aktivität in einer durchschnittlichen Woche erfassen, während Sie mit Ihrem Kind **zuhause** sind. Denken Sie an Aktivitäten, die ***mindestens* *10 Minuten am Stück*** andauern.

*Bitte machen Sie nur eine Angabe. Schwanken Sie zwischen zwei Werten, entscheiden Sie sich bitte für einen dieser Werte.*

| **9. An wie vielen Tagen einer durchschnittlichen Woche, während Sie mit Ihrem Kind zuhause sind, üben Sie etwas anstrengende* Aktivitäten aus?** | **Wie lange sind Sie an diesen Tagen durchschnittlich körperlich aktiv?** |
| --- | --- |
| **Bei diesen Aktivitäten kommt man leicht außer Atem und etwas ins Schwitzen, aber man kann sich während der Belastung noch unterhalten: „Laufen, ohne zu schnaufen“. Allerdings ist diese Form der Bewegung deutlich anstrengender als eine Alltagsaktivität. Beispiele: Radfahren, Schwimmen bei gemütlicher Geschwindigkeit oder Walken. Hierzu zählt nicht das zu Fuß gehen (z.B. Spaziergang).* | |
| - An keinem Tag - Anzahl der Tage (1-7):   Um welche Aktivität/en handelt es sich dabei? | - 10 Minuten - 15 Minuten - 30 Minuten - 45 Minuten - 60 Minuten - 75 Minuten - 90 Minuten und mehr |
| **10. An wie vielen Tagen einer durchschnittlichen Woche, während Sie mit Ihrem Kind zuhause sind, üben Sie anstrengende** Aktivitäten aus?** | **Wie lange sind Sie an diesen Tagen durchschnittlich körperlich aktiv?** |
| ***Bei diesen Aktivitäten kommt man außer Atem und ins Schwitzen, man kann sich währenddessen kaum noch unterhalten. Beispiele: Schnelles Radfahren, Schwimmen (Bahnen auf Zeit) oder Joggen.* | |
| - An keinem Tag - Anzahl der Tage (1-7):   Um welche Aktivität/en handelt es sich dabei? | - 10 Minuten - 15 Minuten - 30 Minuten - 45 Minuten - 60 Minuten - 75 Minuten - 90 Minuten und mehr |

*(In Anlehnung an die Screeningfragen aus dem CARE for CAYA Projekt modifiziert nach dem IPAQ-SF)*

1. **Ihre körperliche Aktivität im Alltag – Aktuelle Situation im Krankenhaus und zuhause**

Denken Sie bei den folgenden beiden Fragen nun an **Gehstrecken**, die Sie in einer durchschnittlichen Woche für ***mindestens 10 Minuten am Stück*** verrichten, während Sie mit Ihrem Kind **im Krankenhaus** sind. Dies kann Gehstrecken wie bspw. auf dem Stationsflur oder im Freien (z.B. beim Spaziergang, Einkaufen) beinhalten.

| **11. An wie vielen Tagen einer durchschnittlichen Woche, während** **Sie mit Ihrem Kind im Krankenhaus sind, gehen Sie mindestens 10 Minuten am Stück zu Fuß?** *(IPAQ-SF)* | |
| --- | --- |
| Tage pro Woche | - Keine entsprechenden Wege zu Fuß 🡪 **weiter zu Frage 13** |

*Zählen Sie die gesamte* ***Gehzeit****, die mindestens 10 Minuten am Stück andauert, zusammen:* *Geben Sie immer Stunden und Minuten an.*

| **12. Wie viel Zeit verbringen Sie für gewöhnlich an einem dieser Tage mit Gehen?** *(IPAQ-SF)* |
| --- |
| Stunden pro Tag Minuten pro Tag |

Denken Sie bei den folgenden beiden Fragen nun an **Gehstrecken**, die Sie in einer durchschnittlichen Woche für ***mindestens 10 Minuten am Stück*** verrichten, während Sie mit Ihrem Kind **zuhause** sind. Dies kann Gehstrecken daheim oder in der Arbeit, Gehen, um von einem Ort zu einem anderen zu gelangen sowie alles andere Gehen zur Erholung, Bewegung und Freizeit beinhalten.

| **13. An wie vielen Tagen einer durchschnittlichen Woche, während Sie mit Ihrem Kind zuhause sind, gehen Sie mindestens 10 Minuten am Stück zu Fuß?** *(IPAQ-SF)* | |
| --- | --- |
| Tage pro Woche | - Keine entsprechenden Wege zu Fuß 🡪 **weiter zu Frage 15** |

*Zählen Sie die gesamte* ***Gehzeit****, die mindestens 10 Minuten am Stück andauert, zusammen:* *Geben Sie immer Stunden und Minuten an.*

| **14. Wie viel Zeit verbringen Sie für gewöhnlich an einem dieser Tage mit Gehen?** *(IPAQ-SF)* |
| --- |
| Stunden pro Tag Minuten pro Tag |

Die 15. Frage bezieht sich auf die gesamte Zeit, die Sie täglich an einem Werktag (Montag bis Freitag) in einer durchschnittlichen Woche mit **Sitzen** verbringen, während Sie mit Ihrem Kind **im** **Krankenhaus** sind. Dies kann Zeit beinhalten wie Sitzen im Stationszimmer oder bei Untersuchungen Ihres Kindes sowie auch zwischendurch im Elternhaus (z.B. am PC, Handy, beim Lesen oder Fernsehschauen sitzend als auch liegend).

*Zählen Sie die gesamte* ***Sitzzeit*** *zusammen: Geben Sie immer Stunden und Minuten an.*

| **15. Wie viel Zeit verbringen Sie in einer durchschnittlichen Woche täglich an einem Werktag mit Sitzen, während Sie mit Ihrem Kind im Krankenhaus sind?** *(IPAQ-SF)* |
| --- |
| Stunden pro Tag Minuten pro Tag |

Die 16. Frage bezieht sich auf die gesamte Zeit, die Sie täglich an einem Werktag (Montag bis Freitag) in einer durchschnittlichen Woche mit **Sitzen** verbringen, während Sie mit Ihrem Kind **zuhause** sind. Dies kann Zeit beinhalten wie Sitzen am Schreib-/Esstisch, Sitzen oder Liegen vor dem Fernseher und auch Sitzen in einem (öffentlichen) Verkehrsmittel.

*Zählen Sie die gesamte* ***Sitzzeit*** *zusammen: Geben Sie immer Stunden und Minuten an.*

| **16. Wie viel Zeit verbringen Sie in einer durchschnittlichen Woche täglich an einem Werktag mit Sitzen, während Sie mit Ihrem Kind zuhause sind?** *(IPAQ-SF)* |
| --- |
| Stunden pro Tag Minuten pro Tag |

| **17. Insgesamt gesehen, wie stark achten Sie auf ausreichend körperliche Bewegung, während Sie mit Ihrem Kind im Krankenhaus sind?** *(Krug 2013 - DEGS1)* | | | | |
| --- | --- | --- | --- | --- |
| - Sehr stark | - Stark | - Wenig | - Gar nicht | - Teils/teils |

| **18. Insgesamt gesehen, wie stark achten Sie auf ausreichend körperliche Bewegung, während Sie mit Ihrem Kind zuhause sind?** *(Krug 2013 - DEGS1)* | | | | |
| --- | --- | --- | --- | --- |
| - Sehr stark | - Stark | - Wenig | - Gar nicht | - Teils/teils |

**Ende Teil 2**

**Teil 3: Sonstige Fragen – aktuelle Situation**

*Bei den folgenden Fragen bitten wir Sie darum eine Antwortmöglichkeit anzukreuzen und diese kurz im vorgegebenen Feld zu begründen.*

| **19. Weisen Sie seit der Diagnose Ihres Kindes körperliche Beschwerden (z.B. Schmerzen) auf?** | | |
| --- | --- | --- |
| - Ja | - Nein 🡪 **weiter zu Frage 21** | |
| Wenn **Ja,** welche körperlichen Beschwerden haben Sie? | | |
| **20. Bestanden diese Beschwerden auch schon vor der Diagnose Ihres Kindes?** | | |
| - Ja | | - Nein |

| **21. Haben Sie das Gefühl, dass Sie sich seit der Diagnose Ihres Kindes weniger als zuvor bewegen?** | |
| --- | --- |
| - Ja | - Nein 🡪 **weiter zu Frage 22** |
| Wenn **Ja**, was könnten Gründe für Ihr reduziertes Bewegungsverhalten sein? | |
| **22. Haben Sie das Gefühl, dass sich Ihr Kind seit der Diagnose weniger als zuvor bewegt?** | |
| - Ja | - Nein 🡪 **weiter zu Frage 23** |
| Wenn **Ja**, worin sehen Sie Gründe für ein reduziertes Bewegungsverhalten Ihres Kindes? | |

| **23. Erachten Sie es für wichtig, dass sich Ihr Kind während stationärer Aufenthalte, in Abhängigkeit von der Tagesform, bewegt und bspw. an einem betreuten Bewegungsangebot teilnimmt?** | |
| --- | --- |
| - Ja | - Nein |
| Ich finde es wichtig, dass sich mein Kind während stationärer Aufenthalte bewegt, weil…: | Ich finde es nicht wichtig, dass sich mein Kind während stationärer Aufenthalte bewegt, weil…: |

| **24. Erachten Sie es für wichtig, dass sich Ihr Kind während den Phasen zuhause, in Abhängigkeit von der Tagesform, bewegt?** | |
| --- | --- |
| - Ja | - Nein |
| Ich finde es wichtig, dass sich mein Kind zuhause bewegt, weil…: | Ich finde es nicht wichtig, dass sich mein Kind zuhause bewegt, weil…: |

Seit März 2020 bieten wir für die erkrankten Kinder und Jugendlichen (ab dem 3. Lebensalter) ein stationäres Sport- und Bewegungsangebot an. Zukünftig streben wir einen familienorientierten Bewegungsansatz an, weshalb von besonderem Interesse ist, ob Sie sich vorstellen könnten, an einem stationären Bewegungsangebot teilzunehmen.

| **25. Im Falle, dass es ein betreutes Bewegungsangebot für Sie gäbe, während Sie mit Ihrem Kind im Krankenhaus sind, hätten Sie Interesse daran teilzunehmen?** | |
| --- | --- |
| - Ja | - Nein |

| **26. Was sind Gründe, weshalb Sie an einem betreuten Bewegungsprogramm teilnehmen würden?**  *Hier haben Sie die Möglichkeit mehrere Angaben zu machen.* |
| --- |
| - Ablenkung vom Klinikalltag |
| - Es tut meiner Gesundheit gut |
| - Stressreduktion |
| - Einnahme einer Vorbildfunktion gegenüber meinem Kind |
| **Sonstige Gründe:** |
|  |
| **27. Aus welchen Gründen würden Sie ein betreutes Bewegungsprogramm ablehnen?**  *Hier haben Sie die Möglichkeit mehrere Angaben zu machen.* |
| - Ich habe keine Lust mich zu bewegen |
| - Ich bewege mich selbst genügend |
| - Ich bin mit der Betreuung meines Kindes so beschäftigt, dass ich keinen Kopf für meine eigenen Bedürfnisse habe |
| **Sonstige Gründe:** |

| **28. Unter welchen Rahmenbedingungen könnten Sie sich vorstellen an einem betreuten Bewegungsangebot teilzunehmen, während Sie mit Ihrem Kind im Krankenhaus sind?**  *Hier haben Sie die Möglichkeit mehrere Angaben zu machen.* |
| --- |
| **Das Angebot sollte …** |
| - zeitlich flexibel stattfinden. |
| - abwechslungsreich gestaltet sein. |
| - gemeinsam mit meinem Kind stattfinden. |
|  |
| - dann stattfinden, wenn mein Kind währenddessen anderweitig beschäftigt wird (z.B. im Spielzimmer). |
| - **Sonstiges**: |
|  |
| **Mit wem würden Sie ein Bewegungsprogramm gerne durchführen?** |
| - Gemeinsam mit meinem Kind und einer/einem Sporttherapeutin/en |
| - In Einzelbetreuung mit einer/einem Sporttherapeutin/en |
| - Mit anderen Eltern (Gruppenangebot) und einer/einem Sporttherapeutin/en |
| - **Sonstiges:** |
| **In welcher räumlichen Umgebung könnten Sie sich eine Teilnahme vorstellen?** |
| - Im Elternhaus |
| - Im Stationszimmer |
| - Auf dem Stationsflur |
| - In einem Sport- und Bewegungsraum |
| - Im Freien an der frischen Luft |
| **Sonstige Rahmenbedingungen:** |

Gerade in den Phasen zuhause geht es für die gesamte Familie und v.a. für das erkrankte Kind darum sich von der intensiven stationären Behandlung im Krankenhaus wieder zu erholen und wieder Kraft für die nächste stationäre Therapieeinheit zu tanken. Dabei ist es wichtig, dass sich das Kind, sofern es der Gesundheitszustand zulässt, wieder ausgiebig bewegt. Dies gelingt am besten, wenn die ganze Familie dabei mitmacht. In diesem Zusammenhang interessiert uns abschließend, inwieweit wir Sie hinsichtlich Bewegungsangebote für die Phasen, wenn Sie mit Ihrem Kind zuhause sind, unterstützen können, bzw. was Sie sich hierfür wünschen würden.

| **29. Was wünschen Sie sich für die Phasen zuhause hinsichtlich Bewegung für Ihr erkranktes Kind bzw. für die ganze Familie?**  *Hier haben Sie die Möglichkeit mehrere Angaben zu machen.* |
| --- |
| - Trainingsplan mit Übungen für zuhause (z.B. Appgesteuert) |
| - Beratungen zu Bewegungsempfehlungen |
| **Sonstige Wünsche/Anregungen:** |

| **Gibt es Ihres Erachtens wichtige Fragestellungen, die in diesem Fragenkatalog nicht angesprochen wurden?** | |
| --- | --- |
| - Ja | - Nein |
| Wenn **Ja**, welche? | |

**Ende Teil 3 – Ende des Fragebogens**

**Ausgefüllt am: _ _ /_ _ /_ _ _ _**

**Herzlichen Dank für Ihre Teilnahme und Ihre Angaben, damit haben Sie uns sehr weitergeholfen!**
